# A Spectroscopic Investigation of Eu^3+^ Incorporation in *Ln*PO_4_ (*Ln* = Tb, Gd_1-x_Lu_x_, *X* = 0.3, 0.5, 0.7, 1) Ceramics

**DOI:** 10.3389/fchem.2019.00094

**Published:** 2019-02-22

**Authors:** Henry Lösch, Antje Hirsch, Jacqueline Holthausen, Lars Peters, Bin Xiao, Stefan Neumeier, Moritz Schmidt, Nina Huittinen

**Affiliations:** ^1^Helmholtz–Zentrum Dresden–Rossendorf, Institute of Resource Ecology, Dresden, Germany; ^2^Institut für Kristallographie, RWTH Aachen University, Aachen, Germany; ^3^Forschungszentrum Jülich GmbH, Institute of Energy and Climate Research, Nuclear Waste Management and Reactor Safety (IEK−6), Jülich, Germany

**Keywords:** xenotime, PXRD, solid solutions, Eu^3+^ incorporation, TRLFS, grain boundary, ceramics

## Abstract

We have investigated the incorporation of the luminescent Eu^3+^ cation in different *Ln*PO_4_ (*Ln* = Tb, Gd_1−x_Lu_*x*_, *x* = 0.3, 0.5, 0.7, 1) host phases. All samples were analyzed with powder X-ray diffraction (PXRD), Raman spectroscopy, and site-selective time-resolved laser-induced luminescence spectroscopy (TRLFS) directly after synthesis and after an aging time of one year at ambient conditions. The PXRD investigations demonstrate the formation of a TbPO_4_ phase in an uncommon anhydrite-like crystal structure evoked by a pressure-induced preparation step (grinding). In the Gd_1−x_Lu_*x*_PO_4_ solid solution series, several different crystal structures are observed depending on the composition. The TRLFS emission spectra of LuPO_4_, Gd_0.3_Lu_0.7_PO_4_, and Gd_0.5_Lu_0.5_PO_4_ indicate Eu^3+^–incorporation within a xenotime-type crystal structure. TRLFS and PXRD investigations of the Gd_0.7_Lu_0.3_PO_4_ composition show the presence of anhydrite, xenotime, and monazite phases, implying that xenotime no longer is the favored crystal structure due to the predominance of the substantially larger Gd^3+^–cation in this solid phase. Eu^3+^–incorporation occurs predominantly in the anhydrite-like structure with smaller contributions of Eu^3+^ incorporated in monazite and xenotime. The electronic levels of the Eu^3+^–dopant in Gd_0.3_Lu_0.7_PO_4_ and Gd_0.5_Lu_0.5_PO_4_ xenotime hosts are strongly coupled to external lattice vibrations, giving rise to high-energy peaks in the obtained excitation spectra. The coupling becomes stronger after aging to such an extent that direct excitation of Eu^3+^ in the xenotime structure is strongly suppressed. This phenomenon, however, is only visible for materials where Eu^3+^ was predominantly incorporated within the xenotime structure. Single crystals of Eu^3+^–doped LuPO_4_ show no changes upon aging despite the presence of vibronically coupled excitation peaks in the excitation spectra measured directly after synthesis. Based on this observation, we propose a lattice relaxation process occurring in the powder samples during aging, resulting in Eu^3+^ migration within the crystal structure and Eu^3+^ accumulation at grain boundaries or xenotime surface sites.

## Introduction

A large challenge yet to be resolved by many countries is the safe disposal of high-level radioactive waste materials, such as spent reactor fuel and various waste streams from, e.g., fuel reprocessing facilities and dismantled nuclear weapons. Currently, the immobilization of high-level liquid wastes generated in the reprocessing of spent nuclear fuel is done by vitrification in borosilicate glass as a first-generation host for the heterogeneous waste solutions (Donald et al., 1997). Ceramic materials (poly- or single-phase) like synroc (Ringwood, 1982), pyrochlore, perovskite, and rare earth element phosphates (REEPO_4_ or *Ln*PO_4_) represent alternative materials to the borosilicate glass as potential second-generation hosts (Ewing and Wang, 2002; Lumpkin, 2006; Caurant et al., 2007). The naturally occurring *Ln*PO_4_–minerals xenotime and monazite have been shown to contain varying quantities of primordial actinides (U, Th), in some cases up to more than 20 wt–% (Gramaccioli and Segalstad, 1978; van Emden et al., 1997; Seydoux-Guillaume et al., 2004; Förster et al., 2008). These crystalline minerals have existed for millions of years, showing good chemical durability and radiation tolerance. Therefore, xenotime- and especially monazite-based ceramics are regarded as candidate materials for the immobilization of long-lived actinide elements (Ewing and Wang, 2002; Seydoux-Guillaume et al., 2004; Lumpkin, 2006; Oelkers and Montel, 2008; Clavier et al., 2011; Vance et al., 2011; Schlenz et al., 2013; Behnam et al., 2017; Neumeier et al., 2017). The orthophosphates of the rare earth elements crystallize in two different crystal structures, depending on the cation radius. The common oxidation state of the rare earth elements is +3. The larger elements from La to Eu crystallize in the monoclinic monazite structure, while the smaller elements from Ho to Lu and Y form the tetragonal xenotime structure (Mullica et al., 1985; Ni et al., 1995; Boatner, 2002; Clavier et al., 2011; Mesbah et al., 2014; Rafiuddin et al., 2014). The lanthanide-orthophosphates GdPO_4_, TbPO_4_, and DyPO_4_ show polymorph properties depending on pressure and temperature (Celebi and Kolis, 2002; Boakye et al., 2008).

The incorporation of actinides within the *Ln*PO_4_ crystal structures occurs *via* various mechanisms, depending on the oxidation state of the dopant. Trivalent actinides (or actinide analog elements in the lanthanide series) directly substitute at the host cation sites in the crystal structure, while tetravalent or higher-valent actinides require an additional co-dopant to preserve charge neutrality. Such substitution reactions involving tetravalent actinides are, e.g., *An*^4+^ + Ca^2+^ = 2 *Ln*^3+^ or *An*^4+^ + ${\text{SiO}}_{4}^{4-}$ = *Ln*^3+^ + ${\text{PO}}_{4}^{3-}$, where the co-dopants are substituted for two host cations or a host cation and an anion, respectively (van Emden et al., 1997; Förster et al., 2008). In addition, substitution mechanisms involving the formation of host-site vacancies (□_*Ln*_) according to 3 *An*^4+^ + □_*Ln*_ = 4 *Ln*^3+^ have been proposed (Vance et al., 2011).

For the safe disposal of actinide elements within the *Ln*PO_4_ structure, a sound understanding of the incorporation behavior of the actinide dopants is required. Actinide accumulation at grain boundaries or actinide clustering within the solid matrix may lead to unwanted reactions such as segregation of the actinide from the solid structure or incongruent dissolution of the waste matrix when in contact with water. To probe such local incorporations, spectroscopic methods sensitive to the dopant environment can be used. Site-selective time-resolved laser-induced luminescence spectroscopy (TRLFS) is a valuable tool for the determination of dopant site symmetries and short-range order/disorder phenomena within the crystal structure, where luminescent *Ln* or *An* can be used as structural probes in the sample. In this context, Eu^3+^ as an analog for the trivalent actinides Pu^3+^, Am^3+^, and Cm^3+^, is often used as a dopant due to its strong, long-lived luminescence. The fine-structure in the luminescence spectra as a consequence of crystal field perturbations around incorporated Eu^3+^ as well as the relative intensities of the emission transitions can be used to gain insight into the local structure in terms of, e.g., the site symmetry in the host lattice (Binnemans, 2015).

In our previous work, we conducted laser spectroscopic investigations of Eu^3+^ incorporation in a series of monazite end-members, (LaPO_4_–GdPO_4_, excluding redox-sensitive CePO_4_ and radioactive PmPO_4_) (Huittinen et al., 2016). Based on the luminescence emission behavior of the Eu^3+^ dopant, such as the half—width of the Eu^3+^ excitation peak and the ^7^F_2_/^7^F_1_ emission peak ratio, we could show that a slight distortion of the monazite crystal lattice around the Eu^3+^ dopant occurs, when going from very similar host and dopant cation radii (such as Eu^3+^ doped in GdPO_4_) toward larger differences (Eu^3+^ doped in LaPO_4_). Despite this small lattice distortion, however, a perfect substitution of the Eu^3+^ dopant for the host cation sites in all investigated monazites was obtained, independent of the size of the host cation. By extending the study to higher dopant concentrations in investigations of a series of La_1−x_Gd_*x*_PO_4_ solid solutions (Gd^3+^ serving as trivalent dopant analog) co-doped with 500 ppm Eu^3+^, a disordering of the monazite solid solution series due to a broader distribution of *Ln*···O bond distances in the mixed solids was observed. However, no preferential incorporation of the Eu^3+^ dopant on host cation sites with similarly sized cation radii (Gd^3+^ rather than La^3+^) could be detected, speaking for the use of monazites as host matrices for the immobilization of actinides with varying cation radii (Luo et al., 2009; de Sousa Filho and Serra, 2011; Huittinen et al., 2016, 2017).

In the present work, we have extended the investigations of Eu^3+^ incorporation in lanthanide phosphates to comprise the smaller and heavier lanthanide elements synthesized as water-free, high-temperature *Ln*PO_4_ phases predominantly in the xenotime structure. As host cations we investigated the largest (Tb) and smallest (Lu) lanthanide expected to crystallize in the xenotime structure. We also investigated the solid solution row Gd_1−x_Lu_*x*_PO_4_ (*x* = 0.3, 0.5, 0.7, 1) to account for the substituent concentrations (*x*) resulting in xenotime solid solutions rather than monazite or mixed-phase compounds. In analogy with our previous studies, we used a small quantity of Eu^3+^ (500 ppm) as a luminescent probe in the solid phases to avoid possible metal-metal concentration quenching effects in the materials (Huittinen et al., 2016, 2017). Thus, in the xenotime solid solution series (Gd_1−x_Lu_*x*_PO_4_), Gd^3+^ is taken as a surrogate for the trivalent actinide elements, while 500 ppm Eu^3+^ serve as local structural probe in the samples. To allow for direct comparison with the monazite studies, the same aqueous synthesis route employed for the synthesis of the highly crystalline monazites was used to obtain the xenotime–type solids in the present work (Huittinen et al., 2016, 2017). The site-selective TRLFS technique was applied to study the distribution of Eu^3+^ in the synthetic xenotime phases, while powder X-ray diffraction (PXRD) and Raman spectroscopy were used for bulk structural investigations. The PXRD and spectroscopic investigations were conducted directly after synthesis as well as after an aging period of 1 year, to account for the stability of the initially formed local coordination environment of the dopant. The aging studies were complemented with spectroscopic results obtained for xenotime single crystals, previously synthesized and studied by our group (Xiao et al., 2018) after a similar aging period of ~1 year.

## Experimental

### Sample Synthesis and Characterization

#### Powder Samples

Xenotime–type *Ln*PO_4_ (*Ln* = Tb, Gd_1−x_Lu_*x*_, *x* = 0.3, 0.5, 0.7, 1) were synthesized according to the same procedure as employed in our previous studies for the synthesis of highly crystalline monazite solids (Huittinen et al., 2016, 2017). Lanthanide nitrate salts were dissolved in deionized water in the desired concentrations and precipitated by slow addition of H_3_PO_4_ according to equation (1):

(1) $$\begin{array}{r} Ln{\left(NO_{3}\right)}_{3}+\;H_{3}PO_{4}\;\overset{500\;ppm\;Eu^{3+}}{\to }\;LnPO_{4}:Eu^{3+}+3\;HNO_{3} \end{array}$$

After 1 week at 90°C, the precipitate was obtained by centrifugation and washed with deionized water to remove nitrate ions. The precursor was dried and milled, followed by calcination (2 h, 600°C) and sintering (5 h, 1450°C) to yield the crystalline xenotime product. The powders were characterized (see description below) and aged under ambient conditions in the laboratory for 1 year.

Phase purity and crystallinity of the synthetic xenotime phases were characterized with PXRD directly after synthesis using a Bruker D4 Endeavor diffractometer with a θ−2θ geometry, CuK_α_ radiation in the 2θ-range 10–100° and a step size of 0.02°. A second PXRD survey was performed 1 year after the synthesis to examine possible changes in the crystal structure on a bulk scale with a Phillips X'Pert Pro diffractometer using a θ−θ geometry, CuK_α_ radiation in the range 10–80° and a step size of 0.008°. For the identification of crystalline phases and their abundance in the synthetic material, Rietveld refinement was performed (TOPAS Academic 5) (Coelho, 2003).

Additionally, Raman measurements of the solid solution series at room temperature were performed on a LabRam ARAMIS (Horiba Jobin Yvon) with an excitation wavelength of 532 nm (Nd:YAG), which was calibrated on a silicon wafer using the first-order Si line at 520.7 cm^−1^. For all measurements, a 1,800 lines/mm diffraction grating was used with a slit of 100 μm, a hole of 300 μm, and a neutral density filter D 0.3 (50% transparency), respectively. The peaks of interest are located between 100 and 1,300 cm^−1^.

#### Single Crystals

Selected xenotime single crystals synthesized in our previous study were investigated with the TRLFS method after an aging time of ~9 months to complement the aging investigations of the powder samples. A detailed description of the synthesis is given in Xiao et al. (2018). Briefly, LuPO_4_ single crystals were obtained by a flux-growth method (Li et al., 2014, 2017). The starting material was a dry powder obtained from polycrystalline precursors and chemicals from Alfa Aesar. For a typical experiment, the initial reactants (polycrystalline xenotime and Na_2_Mo_3_O_10_ flux) were weighed by a molar ratio of 1:50 and mixed in a mortar. The homogenized powders were then transferred to a platinum crucible and heated to 1,300°C inside a tubular furnace (Fuzhou KLST Equipment Co.) in ambient atmosphere. The temperature was kept for 20 h for a complete and uniform reaction (Table 1). After that, the furnace was cooled down slowly at 4°C/h to 870°C for the nucleation formation and crystal growth. Thereafter, the furnace temperature was rapidly (around 20°C/h) cooled to 400°C followed by quenching. Afterwards, the obtained crystals were washed in hot water to remove any remaining flux.

**Table 1:** Conditions for single crystal growth of LuPO_4_ xenotime.

**Crystal**	**Starting material (mass, g)**	**Holding τ(^**°**^C)**	**Holding time (h)**
LuPO_4_	LuPO_4_ (2.00) + Na_2_Mo_3_O_10_ (182.93)	1,300	20

### Description of *Ln*PO_4_ Crystal Structures

**Figure 1:**
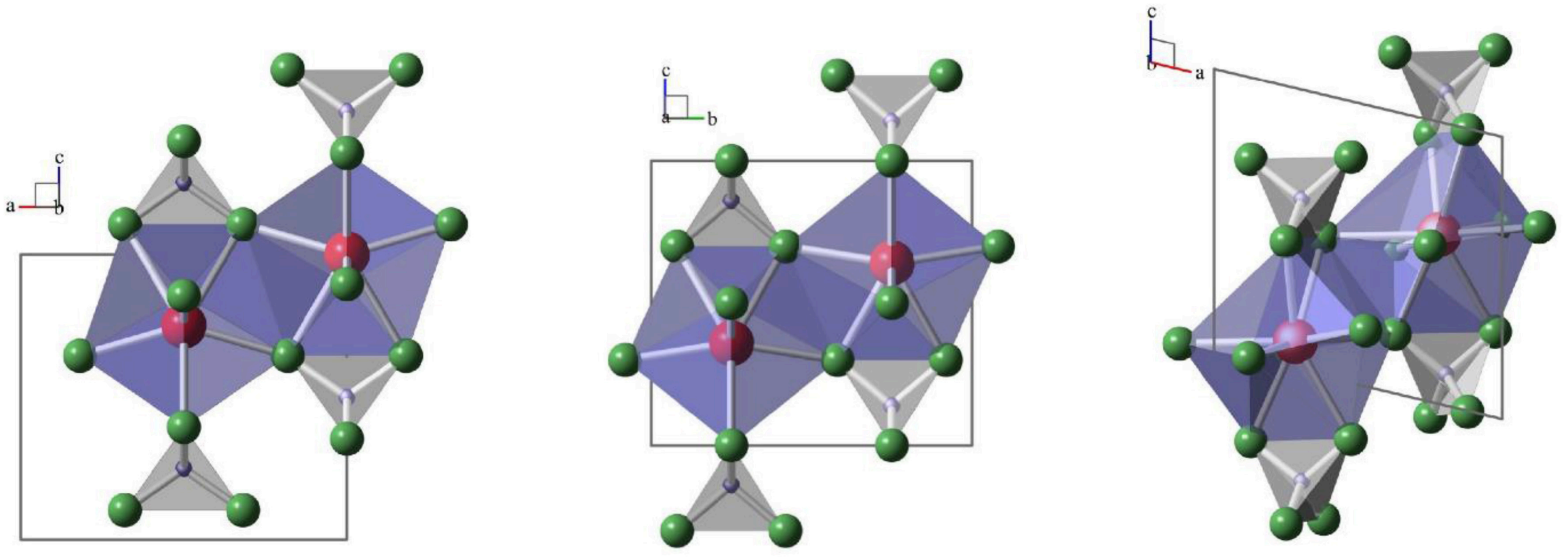
Schematic drawings of [PO_4_]—[*Ln*O_*x*_]—[PO_4_]–chains in xenotime (left, projection along *b*), anhydrite (middle, projection along *a*) and monazite (right, projection along *b*). [PO_4_] are gray tetrahedra with phosphorus-ions in blue and oxygen in green, [*Ln*O_*x*_]–polyhedra are blue with Ln in red.

To understand the spectroscopic data discussed in the present paper, a brief description of the three relevant *Ln*PO_4_ crystal structures and their *Ln*^3+^ site symmetries is given. The main structural feature in all structures, monazite, xenotime, and anhydrite-type is [PO_4_]–[*Ln*O_*x*_]–[PO_4_] chains of edge-sharing [PO_4_]- and [*Ln*O_*x*_]-polyhedra along the *c*-directions of the corresponding unit cells [e.g., (Hay et al., 2013b; Hirsch et al., 2017; Schlenz et al., 2017; Heuser et al., 2018)]. While *x* = 9 in the case of monazite and *x* = 8 for xenotime and anhydrite the similarity of the three structures is striking and can be seen in Figure 1. The [PO_4_]–[*Ln*O_*x*_]–[PO_4_] chains are interlinked via shared oxygen ions of neighboring [*Ln*O_*x*_]-polyhedra in all three structures. The site symmetries for the *Ln*^3+^–cations, however, are rather different, ranging from $\bar{4}2m$ (or *D*_2*d*_) in xenotime, to *m2m* (or *C*_2*v*_) in anhydrite and 1 (or *C*_1_) in monazite. The different site symmetries in the three structure types will give rise to dissimilar Eu^3+^ luminescence spectra, provided that structural incorporation of Eu^3+^ occurs in all solid phases, which enables the detection and distinction of Eu^3+^ incorporation in these different environments as discussed below in section Time–Resolved Laser–Induced Luminescence Spectroscopy, (TRLFS).

### Time-Resolved Laser-Induced Luminescence Spectroscopy, (TRLFS)

Site-selective luminescence spectroscopy is a strong tool for the investigation of the Eu^3+^ environment in crystalline solids. With this method, it is possible to obtain information about the number of non-equivalent species in the system, their site symmetries and potential quenching entities around the Eu^3+^–ion by recording excitation spectra, emission spectra, and luminescence lifetimes, respectively. In the following chapter, only a short description of measured luminescent parameters is given. For a comprehensive description on Eu^3+^ luminescence spectroscopy, the reader is referred to, e.g., (Binnemans, 2015; Huittinen et al., 2016, 2017). For the determination of the number of non-equivalent Eu^3+^–species in the system, the excitation spectra of the ^7^F_0_→^5^D_0_ transition are used. The integration of the Eu^3+^ luminescence intensity as a function of the excitation energy yields peaks in the spectra, which correspond to the number of individual Eu^3+^–species present in the solid. The characteristic emission spectra can be obtained by selective excitation of the individual Eu^3+^–species identified in the excitation spectra. Furthermore, the splitting pattern of emission spectra (typically resolved for the ^7^F_1_– and ^7^F_2_–bands) depends on the site symmetry of the Eu^3+^–ion in the investigated solid. For an incorporation of Eu^3+^ at a cation site in the tetragonal xenotime with site symmetry D_2d_, a 2-and 4-fold splitting of the ^7^F_1_– and ^7^F_2_–bands, respectively, is expected (Milligan et al., 1982; Vance et al., 2011; Binnemans, 2015). By examining the relative intensities of these emission bands, further conclusions can be drawn on the symmetry in the system. The ^5^D_0_→^7^F_1_ transition has a magnetic dipole character. Its intensity is not significantly influenced by the ligand environment. The ^5^D_0_→^7^F_2_ transition, in contrast, has a predominant electric dipole character which is very sensitive to changes in the ligand environment (hypersensitive transition). An increasing relative intensity of the hypersensitive transition results in a larger ^7^F_2_/^7^F_1_ ratio which indicates a decrease of the symmetry in the investigated system (Binnemans, 2015). Finally, the luminescence lifetimes will provide information on quenching entities around the Eu^3+^–dopant. Such quenchers may be hydration water molecules in the first coordination sphere of the luminescent cation, or, e.g., the presence of transition metals or other lanthanides in the sample with available accepting energy levels close to the emitting ^5^D_0_ level of Eu^3+^. In this work, examining high-temperature water-free xenotime phases, due to the sintering step at 1,450°C, hydration water molecules are not expected to be present around the Eu^3+^–dopant. In addition, the host materials were chosen based on their electronic structures, i.e., only trivalent hosts with sufficiently different energy levels from those of Eu^3+^ were chosen (Gd^3+^, Tb^3+^, Lu^3+^). Thus, long luminescence lifetimes above 1.7 ms are expected for Eu^3+^ incorporation in the phosphate hosts according to Equation 2, relating the number of hydration water molecules (*n*) with the measured luminescence lifetime in milliseconds (τ) (Horrocks Jr and Sudnick, 1979; Kimura et al., 1996) (Equation 2).

(2) $$\begin{array}{r} n\left(H_{2}O\right)=1.07\cdot \tau^{-1}-0.62 \end{array}$$

The excitation of the Eu^3+^–ion from the ^7^F_0_ ground state to the emitting ^5^D_0_ excited state was performed directly using a pulsed Nd:YAG (Continuum, Surelite II) pumped dye laser set-up (Radiant Dyes Narrow Scan K). Thereafter, the luminescence light was directed in a polychromator (Andor SR303i) with a 150-, 300-, 600-, or 1,200 lines/mm grating coupled with an ICCD camera (Andor iStar 734) for the recording of the emission spectra 10 μs after the laser pulse in a time window of 10 ms. In every measurement the laser pulse energy and the excitation wavelength were monitored with an optical power meter (Newport 1918–R) and wavelength meter (High Finesse WS−5), respectively. The samples were cooled to ~10 K in a helium-refrigerated cryostat (Cryophysics CCS 100) to achieve the desired spectral resolution.

## Results

### Mineral Phase Composition

**Figure 2:**
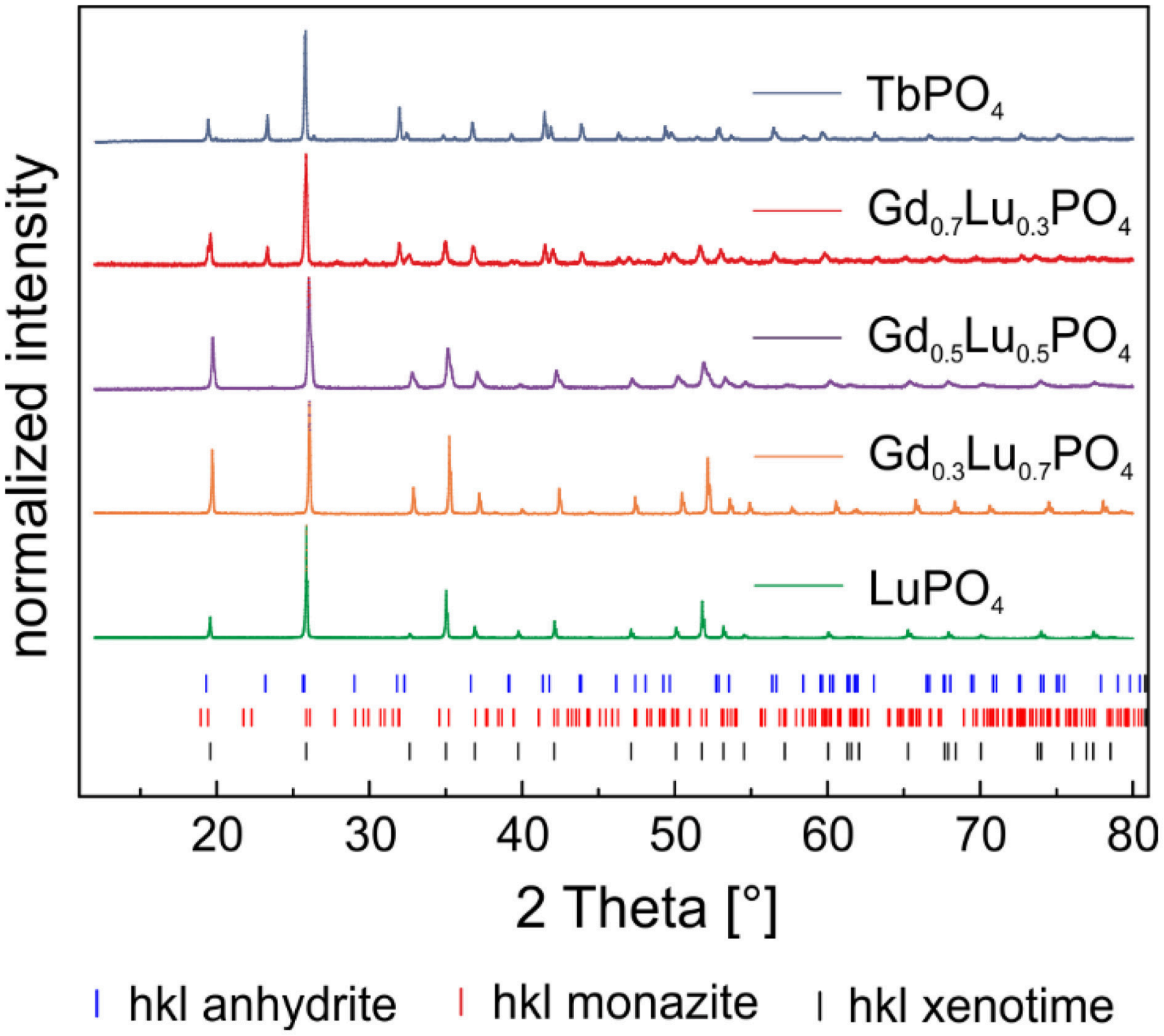
PXRD pattern of Eu^3+^–doped TbPO_4_ and the solid solution series Gd_1−x_Lu_x_PO_4_ (*x* = 0.3, 0.5, 0.7, 1) after 1 year of aging.

Based on the X-ray diffraction pattern of Eu^3+^–doped TbPO_4_ collected directly after synthesis (Supplementary Information 1, blue traces) and after 1 year of aging (Figure 2, blue traces) two different phases can be identified in the sample. TbPO_4_ in the expected xenotime structure (black tick marks) is the minor phase (13 ± 5 wt–%), while the major fraction (87 ± 5 wt–%) could be identified as a TbPO_4_–phase in an uncommon orthorhombic anhydrite-like structure (blue tick marks) after 1 year aging. The anhydrite–type structure, as well as the refinement of the diffraction data is discussed in detail in section TbPO_4_ Anhydrite–Type Structure below. The presence of such an anhydrite-like phase is rather surprising, as pure phase TbPO_4_ xenotime could be obtained in our previous study as precursor for the xenotime single crystals, using the same synthesis procedure as explained above. Small differences, related to the temperature at which the *Ln*PO_4_ precipitation was carried out and powder pre-treatment in terms of grinding, exist between the two syntheses. Here, the synthesis was carried out at 90°C rather than room temperature and the obtained powder was ground twice before and after the heat-treatment. In our previous study, no grinding took place.

The X-ray diffraction patterns of Eu^3+^–doped LuPO_4_, Gd_0.3_Lu_0.7_PO_4_, and Gd_0.5_Lu_0.5_PO_4_ after the synthesis (Supplementary Information 1) and after 1 year of storage (Figure 2, green, orange, and purple traces) are in good agreement with the corresponding ICSD–dataset of LuPO_4_ (ICSD 2505) in the xenotime structure. The PXRD-pattern of Eu^3+^–doped Gd_0.7_Lu_0.3_PO_4_ on the other hand indicates the presence of three different phases in the sample (Supplementary Information 1 and Figure 2, red traces). The major phase can be attributed to an anhydrite-like phase (50 ± 5 wt–%) after 1 year of aging. Similarly to the TbPO_4_ sample, a CaSO_4_ dataset (ICSD 183916) was used for the Rietveld-refinement (Rietveld, 1967) of this phase in the Gd_0.7_Lu_0.3_PO_4_ solid. According to the structure-refinement, ~40 ± 5 wt–% of Gd_0.7_Lu_0.7_PO_4_ is present in the expected xenotime structure, the remaining 10 ± 5 wt–% are monazite (red tick marks). A GdPO_4_–dataset (ICSD 79753) was used for the refinement of the monazite structure. Our results clearly show that xenotime solid solution compositions using Gd and Lu and the aqueous synthesis route employed in the present study can be used for Lu substitutions up to 50%. Between 50 and 70% substitutions, xenotime is no longer the favored crystal structure due to the predominance of the substantially larger Gd^3+^–cation, which has been shown to crystallize in the monazite structure under the aforementioned conditions.

### TbPO_4_ Anhydrite–Type Structure

**Figure 3:**
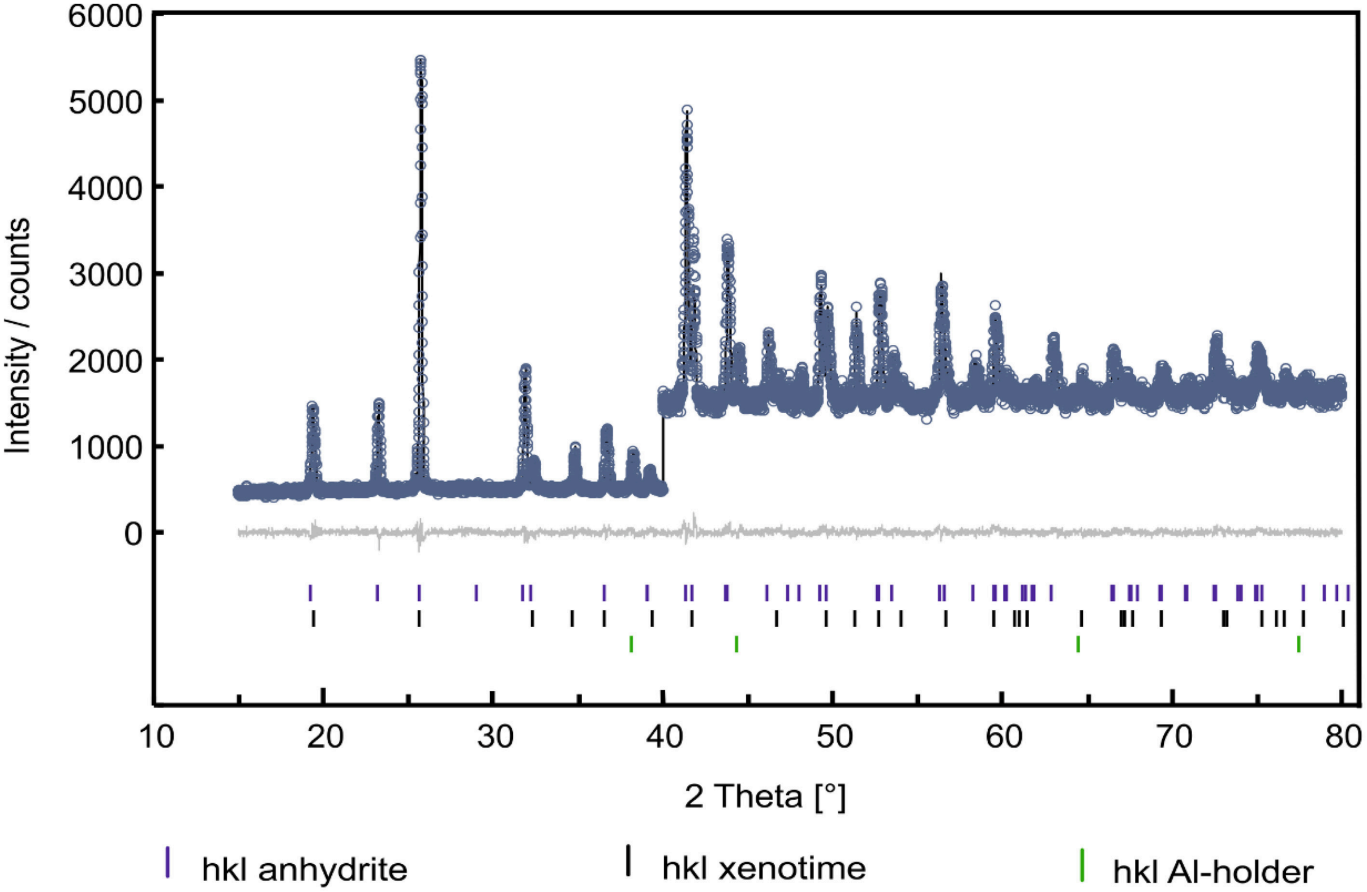
Rietveld-plot of an X-ray diffraction pattern containing ~80 wt.–% of anhydrite-type TbPO_4_ (blue tic-marks), about 15–20 wt–% of the xenotime-type TbPO_4_ black tic-marks) and minor intensities of reflections from the Al–sample holder (green tic-marks). Given are measured intensities (circles), calculated intensities (black line), and the resulting difference curve (gray line around zero on the *y*-axis). From 2θ to 40°, calculated and observed intensities were scaled by a factor of three for the sake of visibility.

The formation of an anhydrite-type phase has previously been observed by Heuser (Heuser et al., 2018) in investigations of a solid solution series of Sm_1−x_Tb_*x*_PO_4_ following a similar synthesis procedure as used in the present work, as well as by Hay et al. for Gd_0.5_Dy_0.5_PO_4_ after synthesis using a fiber push out method (Hay et al., 2013a,b). In Heuser et al. (2018), the formation of the anhydrite-type structure was attributed to mechanical stress induced by, e.g., grinding of the samples. Therefore, in agreement with their work, we interpret the formation of anhydrite-type TbPO_4_ as a result of grinding. Until now, there is no entry in the ICSD database for lanthanide phosphates in an anhydrite-type structure. Thus, for the refinement of this phase, a model of water-free CaSO_4_ in the anhydrite structure in the orthorhombic space group *Bmmb* (e.g., Xu et al., 2017) was used. For anhydrite, also the alternate setting *Amma* (e.g., Antao, 2011) is common. Both settings, however, are no-standard settings of space group number 63, *Cmcm*, see (Aroyo, 2016), with permuted basic vectors. For the refinement, Ca^2+^ was then replaced by Tb^3+^ and the [SO_4_]^2−^-group by a [PO_4_]^3−^-group. A plot of the refinement result using the Rietveld-method (Rietveld, 1967) within the *TOPAS Academic* suite of programs ( e.g., Coelho, 2003) is shown in Figure 3.

In addition to a 6-coefficient background polynomial, individual line profile parameters for the three present phases (TbPO_4_–anhydrite, TbPO_4_–xenotime, Al from the sample holder), lattice parameters, and corresponding fractional coordinates were refined. In order to account for the dominant scattering power of terbium with respect to phosphorous and oxygen, soft bond-valence restraints were introduced for both TbPO_4_–phases. We consider bond-valence restraints much less prescriptive than bond-distance or-angle restraints. The PXRD-data was not of sufficient quality to refine individual atomic displacement parameters. Hence, a small positive value of 0.5 Å^2^ was entered for the isotropic overall displacement parameter and kept constant during the refinement which converged readily, giving an *R*_*wp*_ = 4.38% and *R*_*p*_ = 4.18% with a goodness of fit of *gof* = 1.05.

The resulting crystal structures of both TbPO_4_–anhydrite (*R*_Bragg_ = 1.90%) and TbPO_4_–xenotime (*R*_Bragg_ = 1.81%), are available as Supplementary Material *crystallographic information file (cif)*. The crystal chemical features of TbPO_4_–anhydrite (lattice parameters *a* = 6.9336(2) Å, *b* = 6.9475(2) Å and *c* = 6.1508(2) Å in space group *Bmmb*) essentially correspond to those of anhydrite (CaSO_4_) regarding polyhedra-linkage, however, with P—O bond lengths of 1.53(1)−1.54(1) Å in the slightly distorted [PO_4_]^3−^-tetrahedra (O—P—O angles: 100(1)−112(1)°). The Tb—O bonds in the [TbO_8_]–polyhedra have lengths of 2 × 2.33(1), 2 × 2.35(1), 2 × 2.40(1), and 2 × 2.55(1) Å. All errors stated are uncorrected errors from the correlation matrix of the non-linear least-squares procedure. The close relationship between the anhydrite- and the xenotime-(zircon)-type structures has been reported before (e.g., Hay et al., 2013b), as has the possibility of a stress-induced topotactic, reversible transformation xenotime—anhydrite.

### Local Coordination Environment of Eu^3+^

#### Before Aging

**Figure 4:**
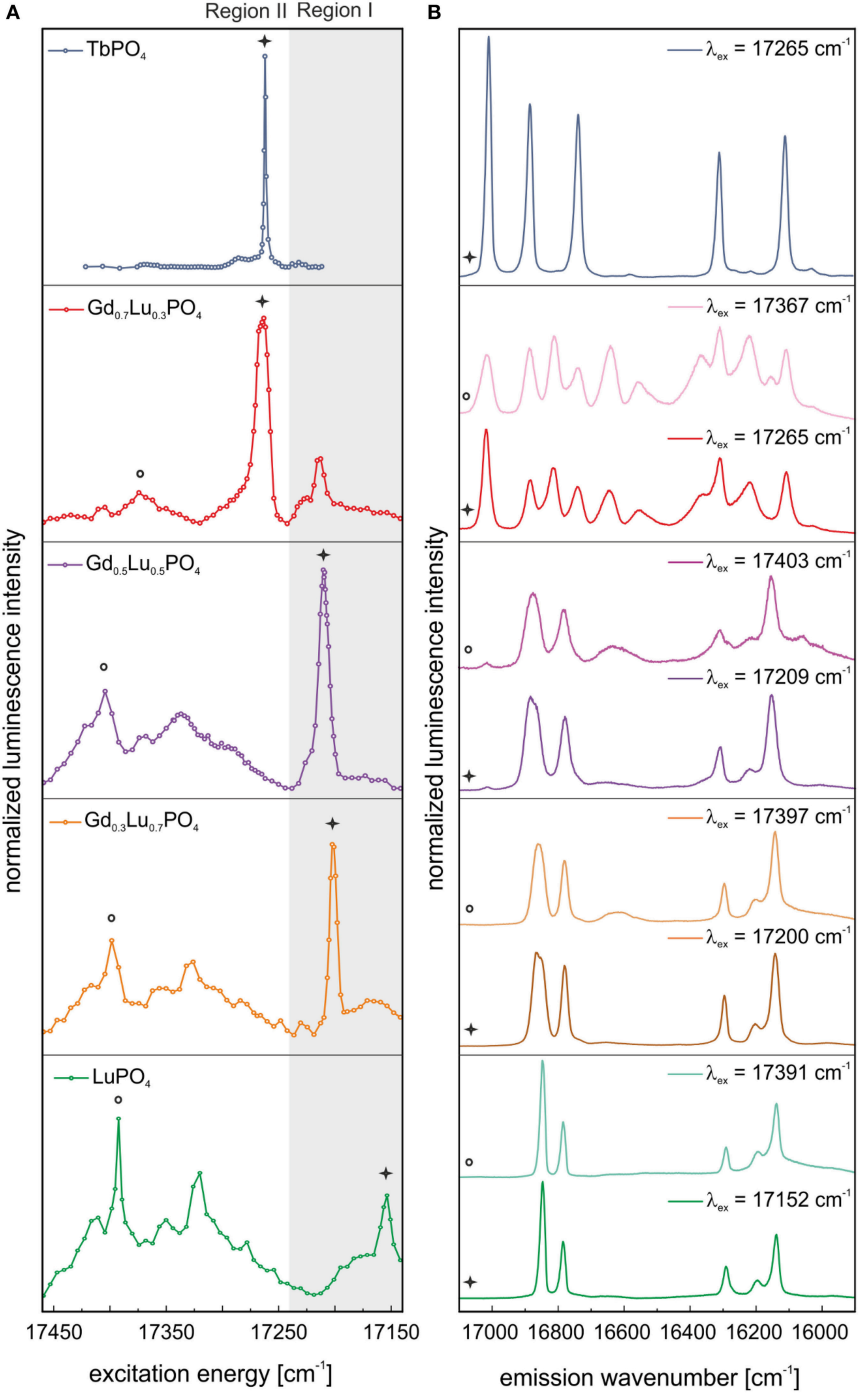
**(A)** Excitation and **(B)** emission spectra of TbPO_4_ and the solid solution series Gd_1−x_Lu_x_PO_4_ (*x* = 0.3, 0.5, 0.7, 1) after synthesis. Spectra are normalized to the overall luminescence intensity.

The excitation spectrum obtained by site–selective TRLFS of Eu^3+^–doped TbPO_4_ before aging is presented in Figure 4A (blue line). One dominant, narrow signal can be identified in the spectrum at 17,265 cm^−1^ (579.2 nm). Through excitation at this excitation peak maximum, the corresponding emission spectrum could be recorded (Figure 4B, blue line). The emission shows a 3- and 2-fold splitting of the ^7^F_1_– and ^7^F_2_–bands, respectively, which does not match the expected splitting pattern of Eu^3+^ incorporated in a xenotime structure [see Time–Resolved Laser–Induced Luminescence Spectroscopy, (TRLFS)]. The 3-fold splitting of the ^7^F_1_–band, i.e., a fully lifted degeneracy by the external crystal field (where the maximum degeneracy is given by 2*J*+1), implies that Eu^3+^ is incorporated in TbPO_4_ at a crystal lattice site with low symmetry. For low symmetries, the ^7^F_2_–band should show a 5-fold splitting. However, this could not be resolved in the obtained spectrum. Three additional very weak lines may be present in the ^7^F_2_–band's spectral range, but their intensity is not sufficient for an unambiguous assignment. The recorded splitting pattern is in agreement with the low-symmetry orthorhombic crystal structure of the TbPO_4_ anhydrite-like phase detected in the PXRD survey. Thus, Eu^3+^ must be incorporated on the Tb^3+^ host cation sites within the anhydrite-like structure. This assignment can be confirmed when comparing the obtained Eu^3+^ emission spectra in the TbPO_4_ powder sample with spectra of Eu^3+^–doped anhydrite (CaSO_4_) from Junot et al. (2014) (Supplementary Information 2). The small differences in the peak positions between both samples arise from the different radii, charge, and bond distances in the cationic and anionic polyhedra.

Excitation spectra of Eu^3+^–doped LuPO_4_ and the “solid solution” series Gd_1−x_Lu_*x*_PO_4_ recorded before aging are presented in Figure 4A (green, orange, purple, and red line). For simplicity, we denote all Gd/Lu samples as solid solutions independent of their phase composition. In comparison to our previous study on monazite *Ln*PO_4_ ceramics (Huittinen et al., 2016), where one narrow Eu^3+^ excitation peak was observed for Eu^3+^–doped *Ln*PO_4_ monazites, a luminescence signal can be detected over the entire examined energy range (17,452–17,152 cm^−1^) for Eu^3+^ incorporation in LuPO_4_, with local maxima at ~17,391, 17,319, and 17,152 cm^−1^ (Figure 4A, green line). The same behavior can be observed for the Gd_0.3_Lu_0.7_PO_4_ and Gd_0.5_Lu_0.5_PO_4_ xenotime solid solutions (Figure 4A, orange and purple line), where the position of the prominent peak present after synthesis is shifted to 17,199 and 17,209 cm^−1^, respectively. For the Gd_0.7_Lu_0.3_PO_4_ sample, multiple crystalline phases were detected in our PXRD studies (50 wt–% of an anhydrite-like phase, 40 wt–% xenotime, and 10 wt–% monazite). Nevertheless, the Eu^3+^ excitation spectrum (Figure 4A, red line) shows the presence of one dominant Eu^3+^–species with an emission peak maximum at 17,265 cm^−1^, which is not accounted for in the other solid solution samples. The peak position is identical to the excitation maximum of Eu^3+^–doped TbPO_4_, implying that the species arises from Eu^3+^ incorporation in either anhydrite or monazite phases, which are not present in the other Gd_1−x_Lu_*x*_PO_4_ compositions.

**Figure 5:**
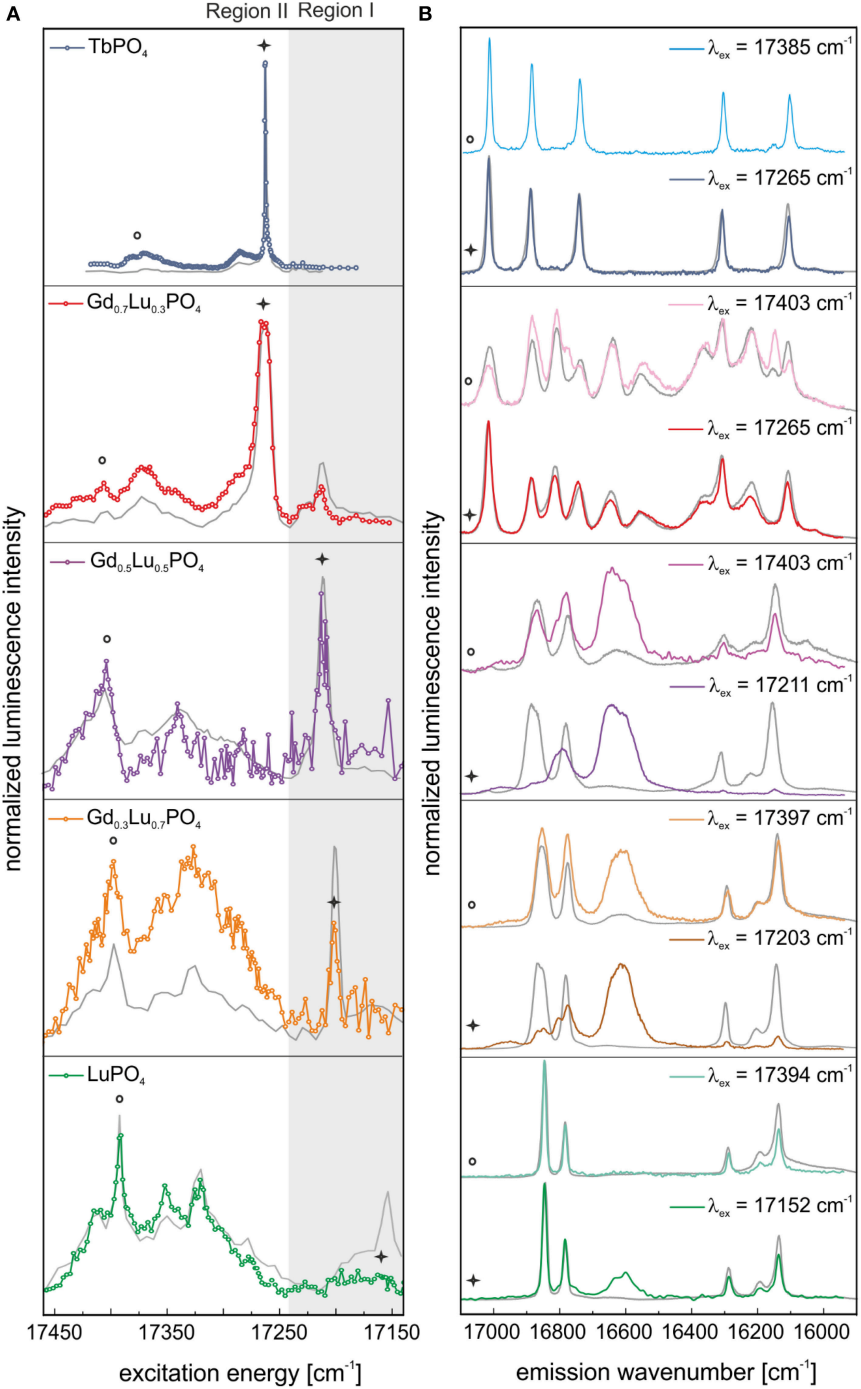
In color: **(A)** Excitation and **(B)** emission spectra of TbPO_4_ and the solid solution series Gd_1−x_Lu_x_PO_4_ (*x* = 0.3, 0.5, 0.7, 1) after 1 year of aging. In gray: the corresponding luminescence data measured directly after synthesis. Spectra are normalized to luminescence intensity.

We can obtain additional information from the emission spectra after selective excitation. To distinguish between the xenotime, monazite, and anhydrite phases, the excitation spectra were divided into two regions:

- Region I: xenotime region (17,240–17,140 cm^−1^), visualized in Figures 4A, 5A with a gray background.
- Region II: high–energy transitions from Eu^3+^–incorporation in monazite, anhydrite, and xenotime, see explanation in the text (>17,240 cm^−1^, white background in Figures 4A, 5A).

Incorporating Eu^3+^ in a xenotime crystal structure should result in a 2– and 4–fold splitting of the ^7^F_1_– and ^7^F_2_–band, respectively, in the emission spectra. This splitting pattern can be observed after selective excitation of the various xenotime solid solutions directly after synthesis, excluding Gd_0.7_Lu_0.3_PO_4_, at the excitation peak maxima in Region I and II, implying that Eu^3+^ substitution for the host cation in these xenotime structures occurs (Figure 4B, green, orange, and purple line). However, as evident in Figure 4B (light orange and light purple), the emission spectra obtained after excitation in Region II show broad features in addition to the narrow emission peaks, which are absent (or nearly absent) in the spectra recorded after excitation in Region I (Figure 4B, orange and purple line). These broad signals may arise from a non-resolved, highly disordered Eu^3+^–species in the sample, or they are vibronic sidebands arising from phonon coupled electronic levels. Further discussion of such electron-phonon coupling is given later in the text. The emission spectra of Gd_0.7_Lu_0.3_PO_4_ show an overlap of different species independent of the excitation wavelength (Figure 4B, red and light red line). In both cases, more than the maximum number of lines (2*J* + 1) is observed, clearly indicating that multiple species are excited simultaneously. Spectral overlap with emission signals collected for Eu^3+^–doped TbPO_4_ suggests that one of the species is likely Eu^3+^ in the anhydrite–like structure, but an unambiguous assignment is not possible. This will be further discussed in connection to the aged samples described below.

#### After Aging

Figure 5 shows the excitation (A) and emission (B) spectra of all samples after 1 year of aging. In all cases, the corresponding spectra of the fresh samples are shown in gray. Excitation spectra for both time steps are normalized to the integrated Eu^3+^ luminescence intensity, as we can assume that Eu^3+^ content in the materials will not have changed. After 1 year of storage, only small differences in the excitation spectra in the high-energy region (Region I) of TbPO_4_ can be observed (Figure 5A, blue line). When exciting in the peak maximum at 17,265 cm^−1^ (579.2 nm), the same 3- and 2-fold splitting in the TbPO_4_ emission spectrum can be observed (Figure 5B, blue line). Next to this, there is a change in the relative intensities between the ^7^F_1_– and ^7^F_2_–band, which reflects changes in the coordination symmetry around the Eu^3+^–ion. For Eu^3+^–doped TbPO_4_ an increase of this ratio can be seen from 0.58 directly after synthesis to 0.66 after 1 year of aging, implying a slightly less symmetric Eu^3+^ surrounding in the aged material. The two minor peaks at higher energies (17,385 and 17,286 cm^−1^) increase in intensity in comparison to the Eu^3+^–anhydrite peak in Figure 5A (blue line) after aging. After excitation at these energies, however, precisely the same spectrum as recorded after excitation in the peak maximum was obtained (Figure 5B, light blue line and Supplementary Information 3). Thus, these peaks cannot be assigned to additional non-equivalent Eu^3+^–species. Despite the presence of 13 ± 5 wt–% xenotime in the TbPO_4_ sample, no indication of Eu^3+^ incorporation on xenotime sites is obtained in the TRLFS investigations, which is a first indication of the disfavored incorporation of Eu^3+^ in xenotime.

After aging, especially LuPO_4_, Gd_0.3_Lu_0.7_PO_4_, and Gd_0.5_Lu_0.5_PO_4_ samples showed a large reduction of the xenotime-peak intensity in Region I (Figure 5A, green, orange, and purple line). Excitation at this energy yields emission spectra with the expected 2 + 4-fold splitting for incorporated Eu^3+^ in the xenotime structure (Figure 5B, green, orange, and purple line). However, an additional peak between the ^7^F_1_– and ^7^F_2_–bands has appeared after the aging process. Because of the electronic structure of Eu^3+^, we expect the occurrence of the magnetic transition ^5^D_0_→^7^F_1_ between 17,064 and 16,666 cm^−1^ and the electric dipole transition ^5^D_0_→^7^F_2_ between 16,393 and 15,873 cm^−1^ (Binnemans, 2015). The observed signal in the emission spectra of these samples is clearly shifted between the ^5^D_0_→^7^F_1_ and ^5^D_0_→^7^F_2_ transitions. As no additional signals in the ^7^F_2_–band are noticeable, we can assume that the visible luminescence of the broad signal between 16,666 and 16,528 cm^−1^ is not arising from the emission of incorporated or adsorbed Eu^3+^, but must be related to exclusion of Eu^3+^ from the xenotime cation site, resulting in lattice displacement/distortion or formation of defect sites in the crystal.

In the excitation spectra of Gd_0.7_Lu_0.3_PO_4_ also small changes in the high-energy region (Region I) can be observed after aging (Figure 5A, red line). We believe that a small amount of Eu^3+^ is incorporated within the xenotime structure in the multi-phase Gd_0.7_Lu_0.3_PO_4_ sample as well. However, even after selective excitation at 17,214 cm^−1^ (580.90 nm, Region I), the emission spectra of at least two species overlap, which does not allow for an unambiguous assignment of the local site symmetry based on the collected luminescence spectra (Figure 5B, light red line). When exciting this multiphase sample at 17,265 cm^−1^ (579.20 nm), i.e., at the excitation peak maximum, the resulting spectrum again shows overlap of multiple species (Figure 5B, red line). Here, however, from a comparison with the emission spectra for Eu^3+^ incorporation in anhydrite (Eu^3+^ in TbPO_4_, Figure 4A, blue line), monazite (Eu^3+^ in GdPO_4_ taken from Huittinen et al. (2016), and xenotime (Eu^3+^ in LuPO_4_, Figure 4A, green line), it is evident that a large amount of the emission signal can be attributed to Eu^3+^ incorporation within Gd_0.7_Lu_0.3_PO_4_ in an anhydrite-like structure (Supplementary Information 4). In addition, peaks belonging to both monazite and xenotime can be observed, implying that Eu^3+^ incorporation occurs in all three phases identified in our PXRD survey. Two of the emission peaks cannot be ascribed to any of the three phases (designated in Supplementary Information 4 with a red box), implying that some additional Eu^3+^–species is present in the sample. These peaks completely vanish after a delay time of 2.5 ms (Supplementary Information 4, blue dashed line), suggesting that this species has a rather short luminescence lifetime.

#### Luminescence Lifetimes

After synthesis, a monoexponential luminescence lifetime of 3,100 ± 170 μs of Eu^3+^–doped TbPO_4_ can be observed (Supplementary Information 5) when exciting at the peak maximum at 17,265 cm^−1^ (579.2 nm). This clearly indicates Eu^3+^ incorporation in the crystal structure without any coordinated water molecules around the Eu^3+^. After the aging period, the luminescence lifetime remains identical within error at 3,300 ± 270 μs also showing a monoexponential decay behavior, confirming Eu^3+^ incorporation into the structure. Excitation of the solid solution samples yields a lifetime of 3,050 ± 150 μs independent of excitation wavelength. The same lifetime has been obtained in our previous study for Eu^3+^ incorporated into LuPO_4_ xenotime single crystals (3,080 ± 200 μs) as well as for the Gd_0.7_Lu_0.3_PO_4_ sample. However, for this sample, a second component can be extracted, i.e., a co-excited species, with a significantly shorter lifetime of around 350 ± 37 μs (Supplementary Information 6a).

The same short–lived component is also present in luminescence lifetimes after excitation in Region II for some samples (Supplementary Information 6b). The average lifetime of this component, considering the fits obtained for all compositions is 350 ± 115 μs. Such a short lifetime points toward the presence of luminescence quenchers close to the Eu^3+^–cation in the sample. Such quenchers could be, e.g., hydration water molecules, or transition metals/*f* –elements in the sample with available accepting energy levels close to the emitting ^5^D_0_ level of Eu^3+^. In the former case, a lifetime of 350 μs would correspond to ~2.5 H_2_O molecules in the first coordination sphere of Eu^3+^. All fitted lifetimes have been summarized in Table 2.

**Table 2:** Fitted luminescence lifetimes for all Eu^3+^–doped samples before and after aging. All lifetimes have been rounded to full 10 μs, to reflect the respective error bars.

**Sample**	**Point of time**	**Lifetime [μs]**	**Region/λ**_**ex**_ **[cm**^**−1**^**]**	**n_**H2O**_**
TbPO_4_	After syn.	3, 100 ± 170	II/17,265	0
	After aging	3,300 ± 270		
Gd_0.3_Lu_0.7_PO_4_	After aging	3, 050 ± 150	II/17,397	0
Gd_0.5_Lu_0.5_PO_4_	After aging	3, 050 ± 150	I/17,211	0
		3, 050 ± 150	II/17,403	0
		350 ± 115		2.5
Gd_0.7_Lu_0.3_PO_4_	After aging	3, 050 ± 150	I/17,214	0
		350 ± 115		2.5
		3, 050 ± 150	II/17,367	0
LuPO_4_	After aging	3, 050 ± 150	II/17,394	0
		350 ± 115		2.5
LuPO_4_ single crystal	Both	2, 600 ± 120	II/17,182	0

#### Aged LuPO_4_ Single Crystals

**Figure 6:**
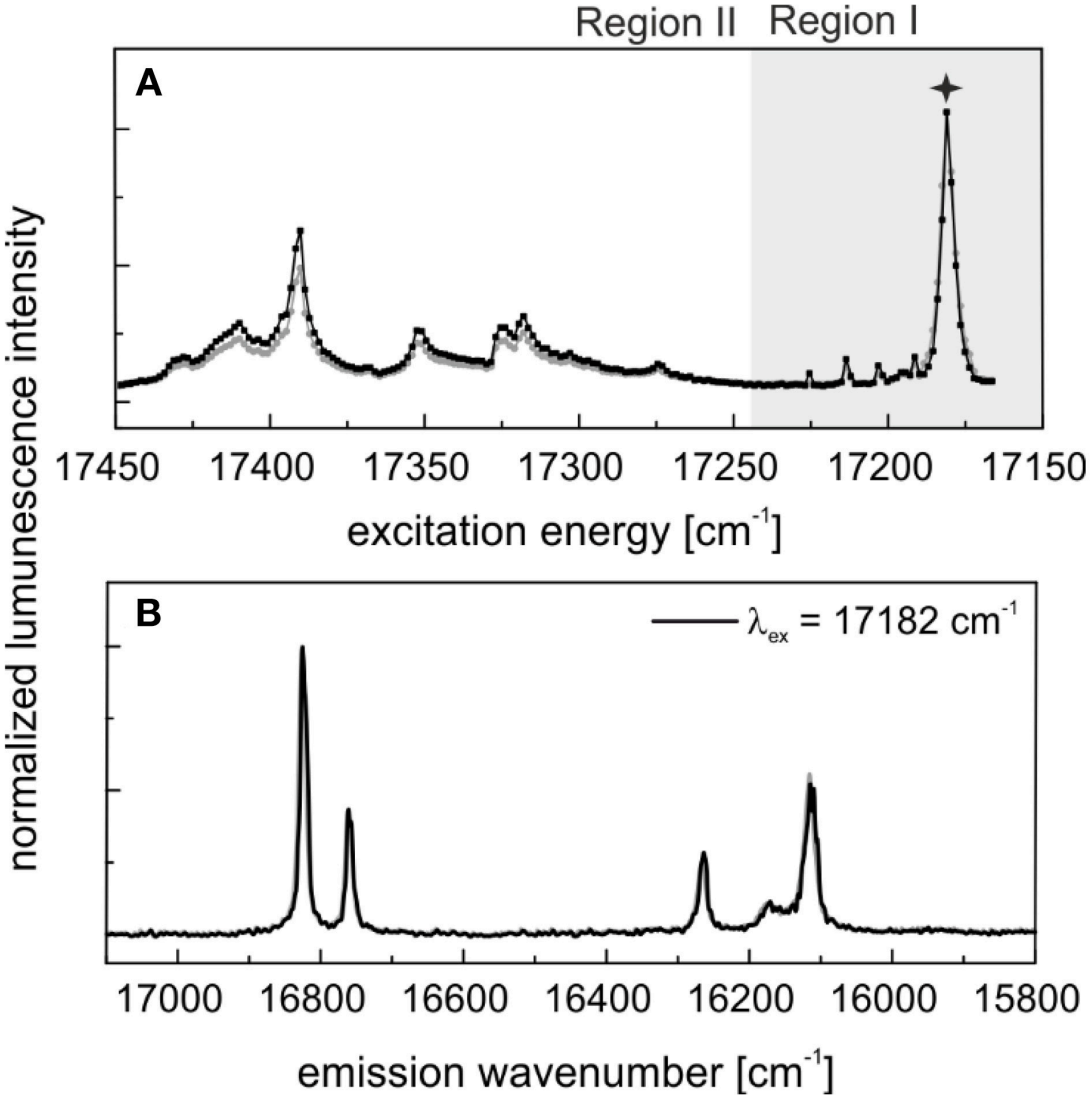
In black: **(A)** Excitation and **(B)** emission spectra of Eu^3+^–doped LuPO_4_ single crystals after 9 months aging. In gray: Corresponding luminescence data measured directly after synthesis [see (Xiao et al., 2018)].

In our previous study (Xiao et al., 2018) we synthesized Eu^3+^–doped LuPO_4_ single crystals based on a flux–growth method. These single crystals were also stored under ambient conditions for a time period of 9 months. Figure 6 shows A) the excitation and B) the emission spectra of the doped LuPO_4_ crystals after the aging period. The spectra obtained directly after synthesis are shown for comparison in gray. In the excitation spectra several signals can be seen in the high–energy region (Region II) next to the main peak at 17,182 cm^−1^ (582 nm). The emission spectra after aging show the expected 2 + 4-fold splitting for Eu^3+^ in a xenotime structure, when exciting at the main peak. In contrast to the powder samples, the aging process has not induced any visible changes in the Eu^3+^ luminescence signal collected from the single crystal sample, a feature that will be discussed in more detail in the discussion section below. For a detailed description of Eu^3+^ incorporation in the single crystal material the reader is referred to Xiao et al. (2018). At both times a lifetime measurement was performed (Supplementary Information 7). They show a monoexponential decay behavior with a lifetime of 2,600 ± 120 μs indicating a complete loss of the hydration-sphere around the europium.

#### Raman Spectroscopic Investigations

**Figure 7:**
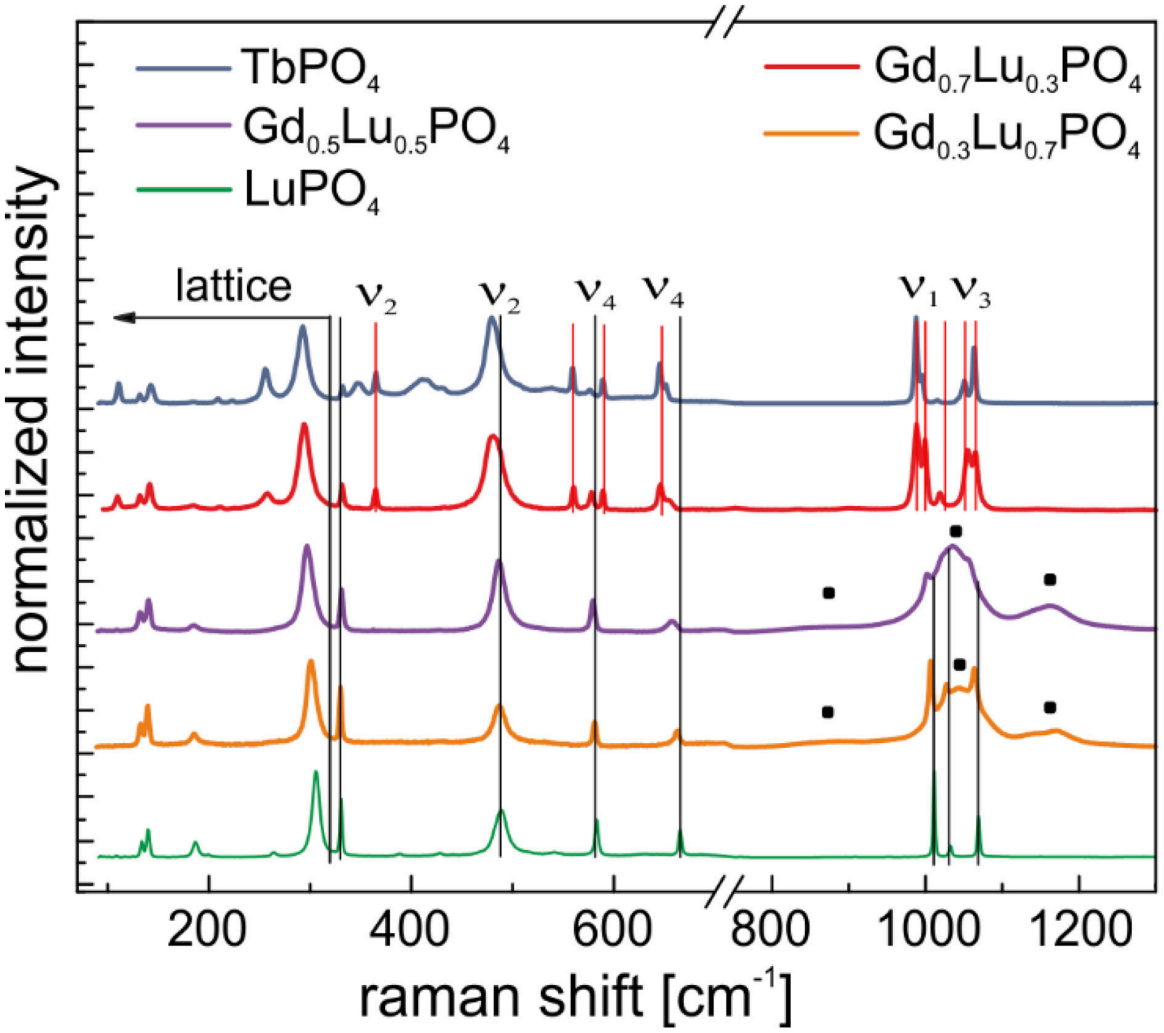
Normalized Raman spectra of the Gd_1−x_Lu_x_PO_4_ solid solution series, LuPO_4_ and TbPO_4_ measured after aging —additional broad peaks in Gd_0.5_Lu_0.5_PO_4_ and Gd_0.3_Lu_0.7_PO_4_. Black lines correspond to xenotime structure, red lines correspond to anhydrite-like structure.

To get a better insight into structural changes occurring in the materials, after aging, where the additional dominant Eu^3+^ emission signal was recorded in our laser spectroscopic investigations, Raman studies were conducted of aged xenotime samples. Figure 7 shows the Raman spectra of the Gd_1−x_Lu_*x*_PO_4_ solid solution series, LuPO_4_, and TbPO_4_ measured after aging. Once again, the spectra can be divided into two regions, Region I between 0 and 300 cm^−1^ with the lattice vibrations [translation of ${\text{PO}}_{4}^{3-}$ (≈150 cm^−1^) and *Ln*^3+^ (≈180 cm^−1^) and external rotational mode of the whole ${\text{PO}}_{4}^{3-}$ tetrahedral unit (≈295 cm^−1^)] and Region II between 300 and 1,300 cm^−1^ with the ν_1_-ν_4_ vibrational normal modes of the ${\text{PO}}_{4}^{3-}$ tetrahedra (Poloznikova and Fomichev, 1994; Yahiaoui et al., 2017). Following the assignment of xenotime Raman bands in Yahiaoui et al. (2017), all four main region bands of the normal vibrational modes (ν_1_-ν_4_) of LuPO_4_, Gd_0.3_Lu_0.7_PO_4_ and Gd_0.5_Lu_0.5_PO_4_ can be assigned (Figure 7, black vertical lines). In the Gd_0.7_Lu_0.3_PO_4_ and TbPO_4_ samples, some additional peaks over the entire spectral range (Figure 7, red vertical lines) are observed, which cannot be assigned to either a xenotime or a monazite phase with the help of literature. For TbPO_4_ in an anhydrite-like structure no reference spectra from the literature are available. Based on this, we tentatively assign these additional signals to TbPO_4_ in the anhydrite-like structure.

In the Gd_0.3_Lu_0.7_PO_4_ and Gd_0.5_Lu_0.5_PO_4_ samples, a significant peak broadening in the symmetric and antisymmetric stretching region (ν_1_ and ν_3_) is observed and three very broad peaks at 880 cm^−1^, 1,040 and 1,168 cm^−1^ emerge in the spectra (Figure 7, black squares). Especially the symmetric and antisymmetric stretching modes, ν_1_ and ν_3_, are highly sensitive to the disordering of the nearest neighbor atoms in the structure (Gouadec and Colomban, 2007). Therefore, this peak broadening as well as the formation of these additional signals indicate a lattice distortion possibly due to Eu^3+^ exclusion from lattice sites into grain boundaries or Eu^3+^ accumulation within the crystal structure. These signals may also be attributed to a beginning phase transformation from xenotime to an anhydrite or monazite phase. Begun et al. (1981) and Tatsi et al. (2008) also observed these additional peaks for TbPO_4_ in a xenotime structure. Tatsi et al. (2008) attributed these signals to electronic transitions of Tb^3+^ due to the pressure sensitivity of these energy levels during a pressure-induced phase transformation. The authors found these additional signals disappear at around 4.5 GPa and re-emerge at around 9.5 GPa, followed by a slow reduction in peak intensity with increasing pressure. After pressure release, the signals reappear. It is possible that Tatsi et al. (2008) indeed observed the pressure-induced phase transition described by Heuser et al. (2018), and consequently these additional signals could be an indication of a lattice distortion as the initial stage of a phase transformation to the anhydrite-type structure.

## Discussion

In the present study, we have combined PXRD, Raman spectroscopy, and site-selective TRLFS investigations to understand the incorporation of Eu^3+^ in *Ln*PO_4_ ceramics predominantly crystallizing in the xenotime structure. Based on our results, the rather large mismatch between the dopant (Eu^3+^) and host (Tb^3+^, Lu^3+^, average cation radii of Gd_1−x_Lu_*x*_PO_4_) cation radii in these phosphates results in a complex incorporation behavior and, in some cases, in the formation of multi-phase solids rather than solid solutions.

Our PXRD results of the end-member TbPO_4_, as well as the Gd_0.7_Lu_0.3_PO_4_ composition, showed the presence of an unexpected and less investigated anhydrite-like structure with minor contributions of the xenotime phase (TbPO_4_) or xenotime and monazite (Gd_0.7_Lu_0.3_PO_4_). Such anhydrite-like *Ln*PO_4_ phases have been reported in e.g., Hay et al. (2013a) investigating Gd_0.5_Dy_0.5_PO_4_ solid solutions using a fiber push-out method for the preparation of the samples. In that study, the authors attributed the formation of the anhydrite–like phase to a pressure effect or thermal stress during the synthesis. Tschauner et al. (2016) investigated the pressure–induced phase transformation in Tb–Gd orthophosphates and postulated a xenotime to monazite transformation path, xenotime (*I*4_1_/*amd*) → anhydrite (*Cmcm*) → distorted anhydrite (*P*2_1_/*m*) → barite (*P*2_1_/*n*) → monazite (*P*2_1_/*n*). Based on this, we suggest a pressure-induced phase transformation in the TbPO_4_ and Gd_0.7_Lu_0.3_PO_4_ samples, leading to the formation of this anhydrite-like phase. This assumption is supported by our previous study on the xenotime single crystal material (Xiao et al., 2018), in which we used the same sample preparation method for the precursor material as reported in this study, but without any grinding steps and without occurrence of an anhydrite-like phase. Based on the luminescence data recorded for Eu^3+^ incorporation in these solids, Eu^3+^ seems to prefer the anhydrite-like or monazite structures over the xenotime one. In TbPO_4_, the presence of Eu^3+^ incorporation in only anhydrite-like orthophosphate could be established, despite the presence of 13 ± 5 wt–% xenotime in this sample. In Gd_0.7_Lu_0.3_PO_4_ Eu^3+^, incorporation could be seen to occur in all three identified phases (anhydrite, xenotime, monazite) with the vast majority of the Eu^3+^ signal originating from an anhydrite-like environment.

The PXRD investigations of the remaining three phases studied in the present work, namely LuPO_4_ and the two solid solutions Gd_0.3_Lu_0.7_PO_4_ and Gd_0.5_Lu_0.5_PO_4_, clearly showed the formation of a single-phased xenotime bulk material. Despite the presence of only xenotime, the luminescence data recorded for Eu^3+^ incorporation in these solid solutions is complex and could be seen to change with aging of the samples, which is something we did not observe for Eu^3+^ incorporation in the two anhydrite-containing phases discussed above. The complex luminescence behavior includes (a) broad excitation signals at high-energies, which, after selective excitation at these energies, yield emission spectra corresponding to Eu^3+^ incorporation in xenotime, (b) biexponential luminescence lifetimes with a short component speaking for quenching phenomena occurring in these samples, and (c) a large emission signal appearing between the ^7^F_1_– and ^7^F_2_–bands in aged samples. Potential reasons for these observations will be discussed below.

### High-Energy Excitation Signals

The high-energy Eu^3+^ excitation signals were observed in all investigated samples, including the multi-phase TbPO_4_ and Gd_0.7_Lu_0.3_PO_4_, samples. However, the latter samples yielded Eu^3+^ emission spectra in an anhydrite-like environment upon excitation in this high-energy region (Region II), while a xenotime signal was obtained for the LuPO_4_, Gd_0.3_Lu_0.7_PO_4_, and Gd_0.5_Lu_0.5_PO_4_, solid solutions. In our previous study, we investigated the incorporation of Eu^3+^ in xenotime single crystals with different host cations (Tb, Y, Ho, Er, Yb, and Lu) using polarization-dependent TRLFS (p-TRLFS) as an analytical tool for the determination of the point symmetry of Eu^3+^. When comparing the recorded excitation spectrum obtained for Eu^3+^–doped LuPO_4_ single crystals measured directly after the synthesis (Xiao et al., 2018) and the corresponding spectrum recorded for the LuPO_4_ xenotime powder directly after synthesis in the present study, the same signals can be seen in both samples (Supplementary Information 8). Based on this knowledge, we can clearly ascertain that these peaks are not related to the synthesis route or to any contamination of the samples.

Excitation peaks at such untypically high excitation energies have previously been observed for Eu^3+^ incorporation in, e.g., scheelite (Xiao and Schmidt, 2017) and powellite (Schmidt et al., 2013). In these studies, the authors attributed the signals to “hot bands” of the major species incorporated in the solid structures arising from an energy transfer process involving the W or Mo host cations. As the host cations of the phosphate ceramics investigated in the present study (excluding Tb^3+^) do not have accepting energy levels in this excitation energy range, a similar hot-band transition mechanism cannot be responsible for the excitation peaks observed in the phosphate hosts.

**Figure 8:**
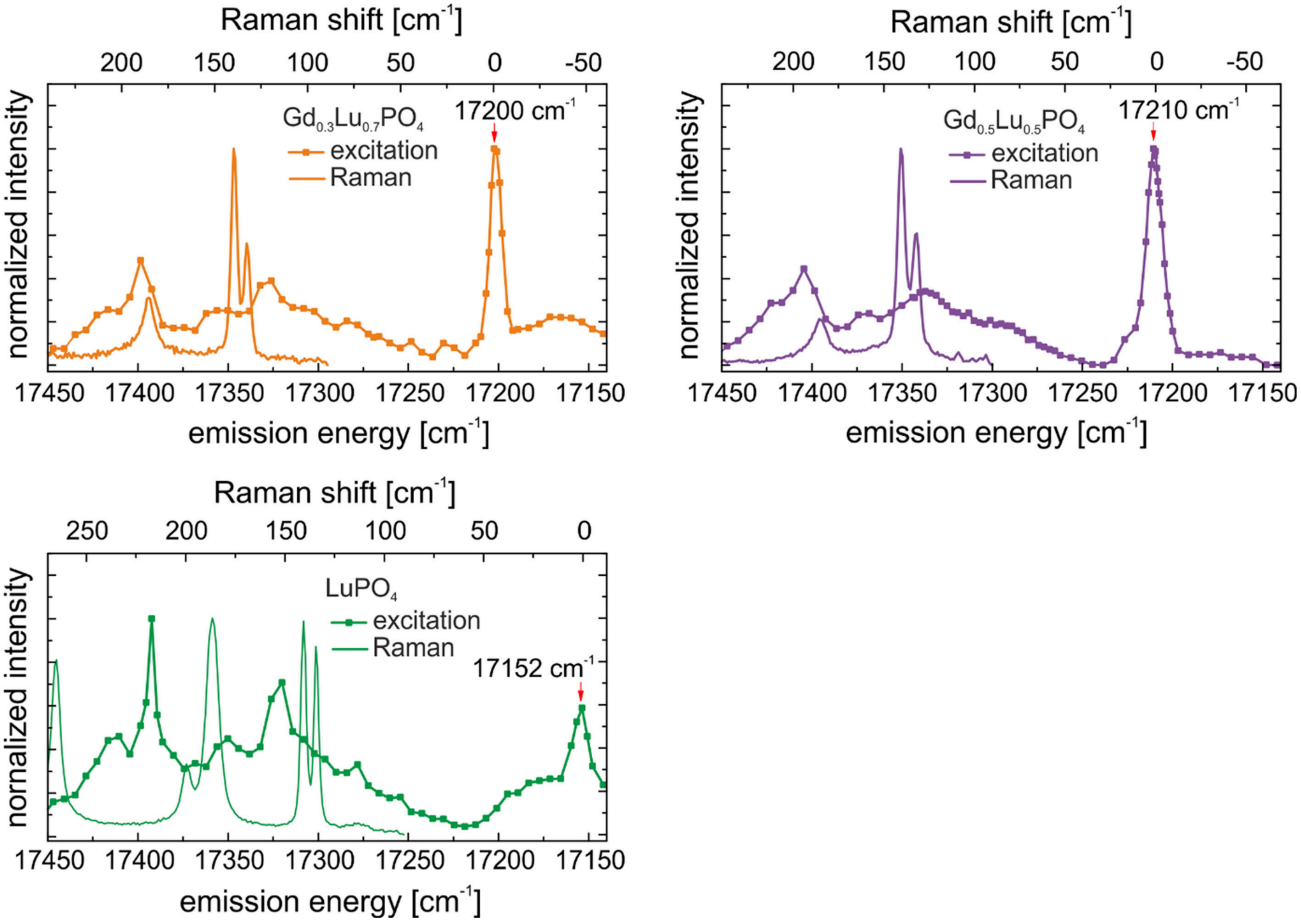
Combined TRLFS excitation spectra after synthesis with mirrored Stokes Raman spectra after 1 year of aging of LuPO_4_, Gd_0.3_Lu_0.7_PO_4_, and Gd_0.5_Lu_0.5_PO_4_.

Thus, following the assignment from our previous study investigating Eu^3+^ incorporation in xenotime single crystals, we propose a phonon-coupled excitation process occurring in the xenotime host phases. To verify such phonon coupling and to better illustrate such processes, measured Raman spectra of the lattice-vibration range for the xenotime solid solutions adjusted to the excitation energy of the TRLFS spectra are compared with the excitation spectra in Figure 8. From this comparison, it becomes evident that there is a great overlap of Raman lattice phonons and the excitation peaks in the high-energy region (Region II) suggesting a co-excitation of Eu^3+^ (^5^D_0_ ← ^7^F_0_) and low energy external translations in the host lattices.

Upon aging, the excitation peaks in Region I corresponding to Eu^3+^ incorporation in xenotime decrease in intensity, especially for the LuPO_4_ and Gd_0.5_Lu_0.5_PO_4_ compositions (see Figure 5A, green and purple line), while the high-energy excitation peaks become yet more pronounced. It appears that laser-induced phonon excitation becomes the primary process occurring in the solids before the remaining laser energy becomes absorbed by the incorporated Eu^3+^–cation. Thus, at high excitation energies, both, phonons, as well as the incorporated Eu^3+^ are excited, while the laser energy required for direct Eu^3+^ promotion to the ^5^D_0_ emitting state (Region I) is too low due to the dissipation of some of this energy by the (multi)-phonon lattice-excitation process, resulting in the absence of this excitation peak in the recorded spectra. We believe, this strong electron-phonon coupling and the influence of aging can be attributed to slow lattice-relaxation processes and relocation of Eu^3+^ within the crystal structure, as discussed below.

### Short Luminescence Lifetimes

**Figure 9:**
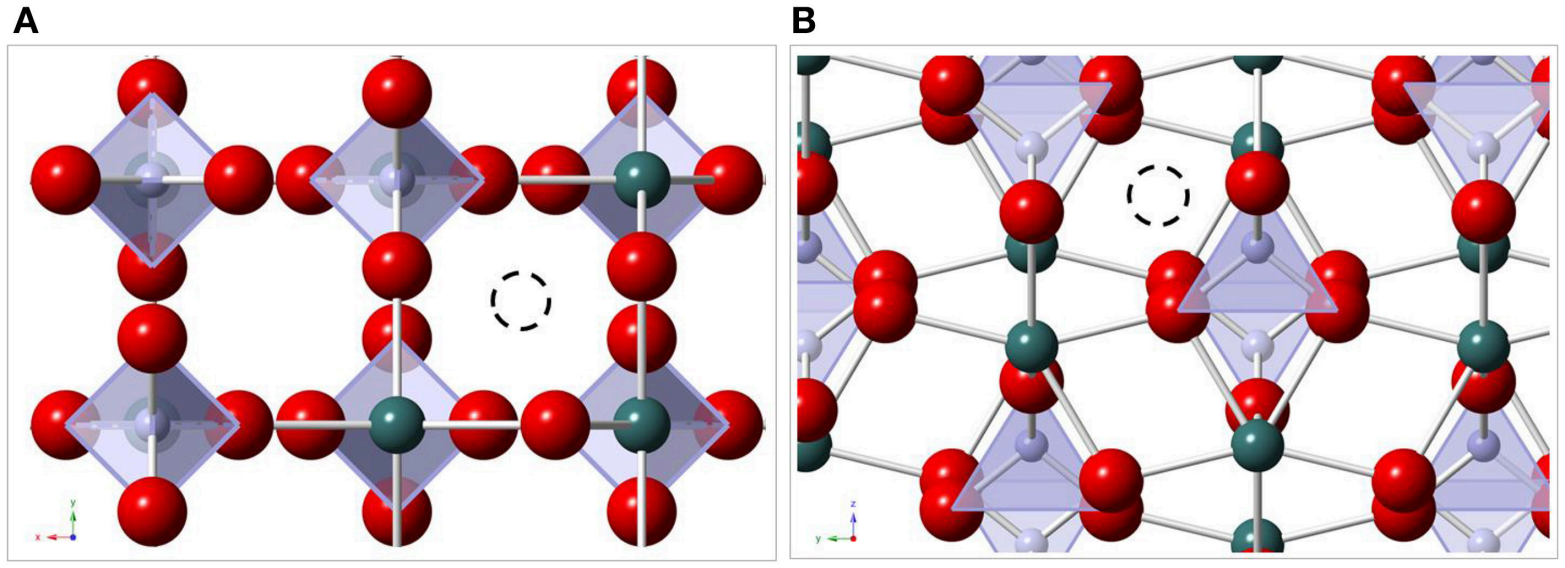
Xenotime crystal structure **(A)** along c-axis, **(B)** along a-axis. red: O^2-^, green: *Ln*^3+^, lila: P^5+^, black dashed: Eu^3+^ in void spaces.

The recorded luminescence-decay curves for the xenotime solid solutions yielded two lifetimes of 3,050 ± 150 and 350 ± 115 μs. The longer lifetime could be assigned to Eu^3+^ incorporation within the xenotime host lattice and it corresponds to a full loss of hydration water around the incorporated dopant cation according to Equation 2. The shorter lifetime, however, is indicative of quenching processes occurring in the sample. The relaxation of an excited electron to the ground state occurs *via* the emission of light with a defined wavelength in dependence of the band gap between the two states. Molecules or atoms with accepting electronic levels close to the ^5^D_0_ emitting level of Eu^3+^ could quench the luminescence lifetime of the Eu^3+^ ion. As already mentioned such quenchers could be other lanthanide cations, neighboring Eu^3+^–ions, and/or water molecules. Due to the filled electron shell of Lu^3+^ cannot act as a luminescence quencher. For Gd^3+^, the accepting energy level is ≈15,000 cm^−1^ above the emitting level of Eu^3+^ (Bünzli and Piguet, 2005). Therefore, an energy transfer from Eu^3+^ to the host cations can be excluded and we have to consider either vibrational quenching by coordinating water molecules or self-quenching processes between adjacent Eu^3+^–cations. For the latter mechanism to be possible, the formation of Eu^3+^–clusters within the phosphate ceramics is required, as the low concentration of 500 ppm Eu^3+^ homogenously distributed within a xenotime structure is much too low for such self-quenching to occur. If Eu^3+^–clusters are present within the solid solution phase due to inadequate incorporation, one would expect either Eu_2_O_3_ or EuPO_4_ monazite to form within the solid solution upon sintering, which again have distinct luminescence emission signals that we could not observe in the present study. Thus, we must explore the presence of hydration water molecules as a potential reason for the short Eu^3+^ lifetime of 350 μs. This lifetime corresponds to ~2.5 H_2_O entities around the Eu^3+^–cation, which is consistent with a partially surface-associated Eu^3+^–species retaining some of its hydration sphere in the adsorption process. Such partial surface association could occur if Eu^3+^ accumulates at grain boundaries due to exclusion from the xenotime crystal structure. Grain-boundary accumulation at the surface in contact with atmospheric humidity over the aging period would allow for water adsorption on the surface and subsequent formation of O···Eu···OH_2_ bonds. This again explains the presence of water around the Eu^3+^−cation despite the previous sintering of the samples at more than 1,400°C. Exclusion of Eu^3+^ from the crystal structure would require some means for Eu^3+^ to migrate through the crystal lattice leading to the accumulation of the cations at grain boundaries. The xenotime structure exhibits unoccupied channels along the *c*-axis of the structure based on edge-connected chains of anion and cation polyhedra. There are two different void spaces in the lattice, one with a tetrahedral geometry within the channels (Figure 9A) and one with a distorted octahedral geometry (Finch and Hanchar, 2003) (Figure 9B). Especially the void space illustrated in Figure 9A is large enough to host a Eu^3+^–cation and enable diffusion of Eu^3+^ through the crystal lattice followed by accumulation at grain boundaries/surface sites. A further validation of our hypothesis of Eu^3+^ migration and accumulation at grain boundaries is obtained from the recorded lifetimes of our single crystals, where one lifetime of >2,500 μs was observed, with no indication of a shorter component at any stage of the aging process. As single crystals possess only minor defects sites and no grain boundaries, only surface sites are available for the exclusion of Eu^3+^ from the host lattice. In addition, the well-formed crystal lattice, obtained in a flux–grow synthesis, hampers the movement of Eu^3+^ leading to less defined environments and a greater distortion around the Eu^3+^–ion. Therefore, the possible space for Eu^3+^ movement from the too small host cation sites is strongly restricted in single crystals.

### Emission Signal Between the ^7^F_1_ and ^7^F_2_ Bands

An open question still to be addressed is the origin of the additional signal between the ^7^F_1_– and ^7^F_2_–band in the emission spectra of Eu^3+^–doped LuPO_4_ and the solid solution series Gd_1−x_Lu_*x*_PO_4_. The signal is very weak or completely absent in the emission spectra recorded directly after synthesis while becoming pronounced or even the most intense signal in some compositions after aging (see Figure 5B).

In Piriou et al. (1997) such additional signals between the ^7^F_1_– and ^7^F_2_–bands (around 16,550 cm^−1^, 604 nm) were observed in site-selective luminescence investigations of Eu^3+^–doped lanthanum disilicate (La_2_Si_2_O_7_) and mullite (2Al_2_O_3_·SiO_2_). The authors described these additional features with a shortening of the Eu···O bond distances and the consequently more covalent bond character, leading to a strong anisotropic crystal field and a large splitting of the ^7^F_1_–band. In our study, the Eu···O bond distance, especially in LuPO_4_, is significantly shorter than the preferred bond distances of Eu···O in EuPO_4_, which is also evident in the extreme bathochromic shift of the ^5^D_0_ transition. An important point is that directly after the synthesis no additional signals were observed in the emission spectra, meaning that these peaks must be related to the presumed exclusion of Eu^3+^ from the crystal structure and migration to the grain boundaries during aging.

We propose two explanations for this unusual luminescence. Firstly, the formation of a vacancy or defect site as a result of the exclusion of Eu^3+^ from an original lattice site. Such vacancy formation will be accompanied by unsaturated oxygen bonds that may further result in delocalized or mobile electrons. Therefore, the electron could interact with incorporated Eu^3+^ or the host cations Lu^3+^/Gd^3+^ resulting in a transition between unoccupied orbitals. Secondly, Eu^3+^ luminescence of Eu^3+^ in void spaces during the migration through the crystal structure could also lead to additional signals in the emission spectra. The narrow void space forces an overlap between Eu^3+^ and oxygen atomic orbitals, strongly influencing the ligand field, subsequently resulting in an extreme splitting of the ^7^F_1_–band. In flux grown LuPO_4_ single crystals the defect density is significant lower than in powder samples. This hampers the migration of Eu^3+^ in the lattice and the formation of defect sites. Our investigations cannot clearly distinguish between these mechanisms and additional explanations may be equally suited to explain our observations. Independent of the specific mechanism, the occurrence of this anomalous luminescence signal after a relatively short storage time under ambient conditions must be related to a segregation of Eu^3+^ from the crystalline lattice.

## Conclusion

The investigations of Eu^3+^–doped *Ln*PO_4_ in the xenotime structure by PXRD, Raman spectroscopy, and site-selective TRLFS provide molecular insights of the incorporation of Eu^3+^ as a chemical analog of the trivalent actinides in a possible ceramic host material for radioactive waste disposal. In contrast to the monazites, where a perfect substitution of Eu^3+^ on host lattice sites occurs independent of the host cations in the structure or the solid solution composition, a much more complex incorporation behavior is found for the smaller lanthanide hosts. We have shown that TbPO_4_ can form an uncommon anhydrite-like phase as a result of grinding during sample synthesis and that Eu^3+^ incorporation occurs in this structure. The pure xenotime solid solutions LuPO_4_, Gd_0.3_Lu_0.7_PO_4_, and Gd_0.5_Lu_0.5_PO_4_, clearly incorporate the Eu^3+^ dopant on host lattice sites, however, segregation of Eu^3+^ from the crystal structure seems to take place over time, resulting in Eu^3+^ accumulation at grain boundaries. This dislocation of the luminescent cation leads to the appearance of emission signals between the ^7^F_1_– and ^7^F_2_–bands, which cannot be assigned to any Eu^3+^ phases but could be related to the formation of defect sites and subsequent delocalized electrons in the crystal structures. Finally, no solid solutions are obtained for the Gd^3+^–rich Gd_0.7_Lu_0.3_PO_4_–composition, but instead a multi-phase solid consisting of anhydrite, xenotime, and monazite is formed. In this multi–phase solid, Eu^3+^ incorporation was found to mainly take place in the anhydrite-like phase.

In summary, the preference of Eu^3+^ for the anhydrite structure, the complex phase mixture obtained at the highest doping level of Gd^3+^, and the segregation of Eu^3+^ to grain boundaries after relative short aging in the xenotime materials, indicate that xenotime ceramics will not serve as a suitable waste form for trivalent actinides from high-level nuclear waste. The presence of multiple phases formed by applying external pressure on the solid material or due to a dopant-to-host structure mismatch will largely limit the predictability of the ceramic's performance during long-term storage. One critical point in this context is the corrosion resistance of such multi-phase materials. Even though pure monazite or xenotime host—matrices have been shown to be resilient toward dissolution, it is unknown how such multi-phase solids will react when in contact with water. Furthermore, the phase transition between the anhydrite structure and the common monazite/xenotime structures is currently not well-understood. Especially the behavior of the dopant during such phase transformation should be investigated in detail to be able to account for the composition (formation of solid solutions vs. multi-phase solids) of the thermodynamically stable solid structures formed after such a transformation. Finally, any conclusions from analog experiments with *Ln*^3+^ must be complemented with results obtained for actual actinide-doped compounds, as the larger mismatch between the actinide dopant cations and the small xenotime hosts may enhance the structural partitioning effects observed for Eu^3+^ in the present study.

## Author Contributions

NH and SN coordinated the experiments. JH performed the sample preparation. LP and AH supported the PXRD experiments. HL and NH performed the TRLFS and Raman investigations and wrote the manuscript with inputs from all co–authors. BX and MS planned and executed the single crystal experiments. All authors have read and approved the paper.

### Conflict of Interest Statement

The authors declare that the research was conducted in the absence of any commercial or financial relationships that could be construed as a potential conflict of interest.
