# Supplementary material for: A Spectroscopic Investigation of Eu3+ Incorporation in LnPO4 (Ln = Tb, Gd1-xLux, X = 0.3, 0.5, 0.7, 1) Ceramics
Source: Front Chem. 2019 Feb 22;7:94. doi: 10.3389/fchem.2019.00094 (PMC6395394; doi:10.3389/fchem.2019.00094)
Supplement: Supplementary file 1 [file Data_Sheet_1.docx]

**Supporting Information**

**A spectroscopic investigation of Eu^3+^ incorporation in *Ln*PO_4_ (*Ln* = Tb, Gd_1–x_Lu_x_, x = 0.3, 0.5, 0.7, 1) ceramics**

H. Lösch^1^, A. Hirsch^2§^, J. Holthausen^3ǂ^, L. Peters^2^, B. Xiao^1^, S. Neumeier^3^, M. Schmidt^1^, N. Huittinen^1*^

^1^Helmholtz–Zentrum Dresden–Rossendorf, Institute of Resource Ecology, Bautzner Landstraße 400, 01328 Dresden, Germany

^2^RWTH Aachen, Institut für Kristallographie, Jägerstraße 17–19, 52066 Aachen, Germany

^3^Forschungszentrum Jülich GmbH, Institute of Energy and Climate Research, Nuclear Waste Management and Reactor Safety (IEK–6), 52425 Jülich, Germany

^§^Current address: Fraunhofer Technology Centre Semiconductor Materials THM, Branch of the Fraunhofer Institute for Integrated Systems and Device Technology IISB, Department Materials, Group Silicon, Am St.–Niclas–Schacht 13, 09599 Freiberg/Sachsen, Germany

^ǂ^Current address: ASK Chemicals GmbH, Reisholzstraße 16−18, 40721 Hilden, Germany

*Corresponding author :

Nina Huittinen

email : n.huittinen@hzdr.de

phone : +49 (0351) 260 2148

fax : +49 (0351) 260 13233

This Supporting Information contains 6 pages and 8 figures


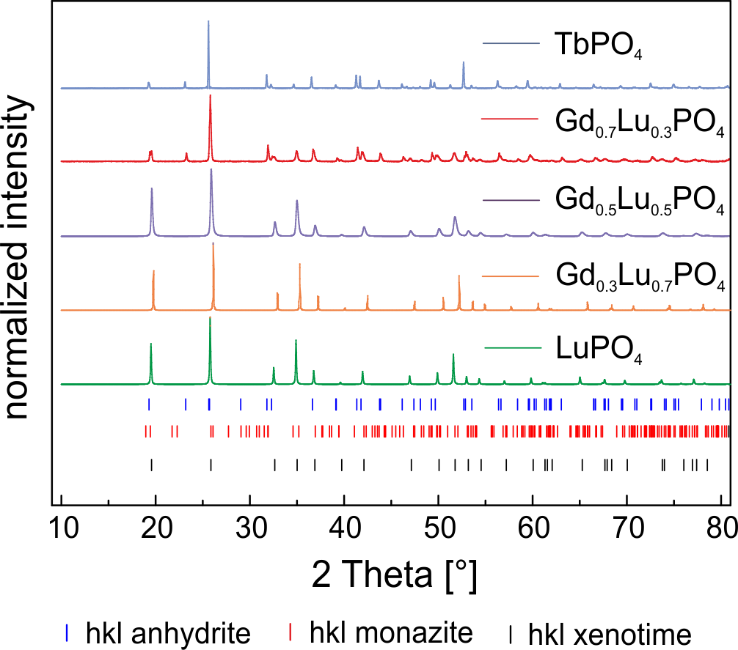


SI 1: PXRD patterns of Eu^3+^–doped TbPO_4_ and the solid solution series Gd_1–x_Lu_x_PO_4_ (x = 0.3, 0.5, 0.7, 1) after synthesis.


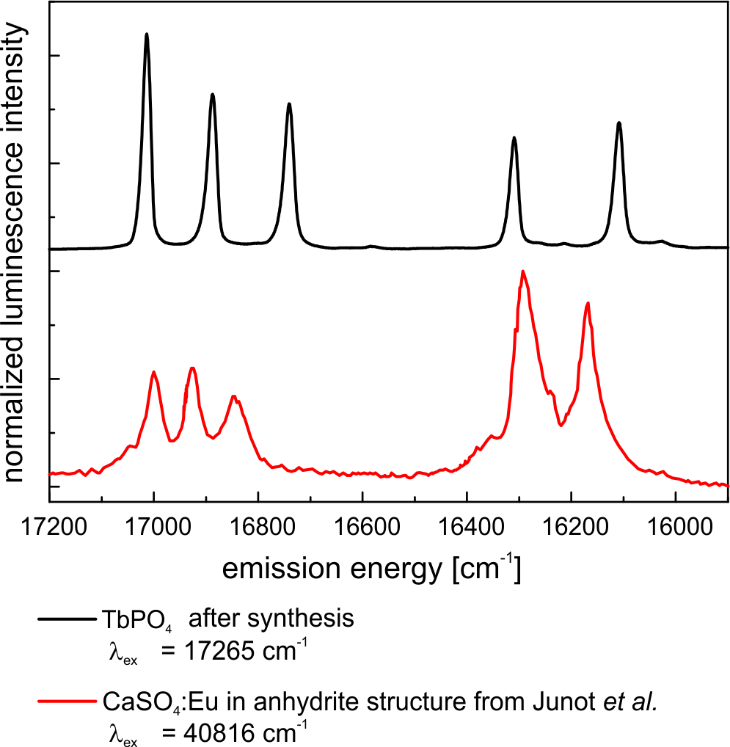


SI 2: Comparison of emission spectra of Eu^3+^–doped CaSO_4_ anhydrite, λ_ex_ = 245nm (replot from (Junot *et al.*, 2014)) and Eu^3+^–doped TbPO_4_ in the anhydrite–like structure.


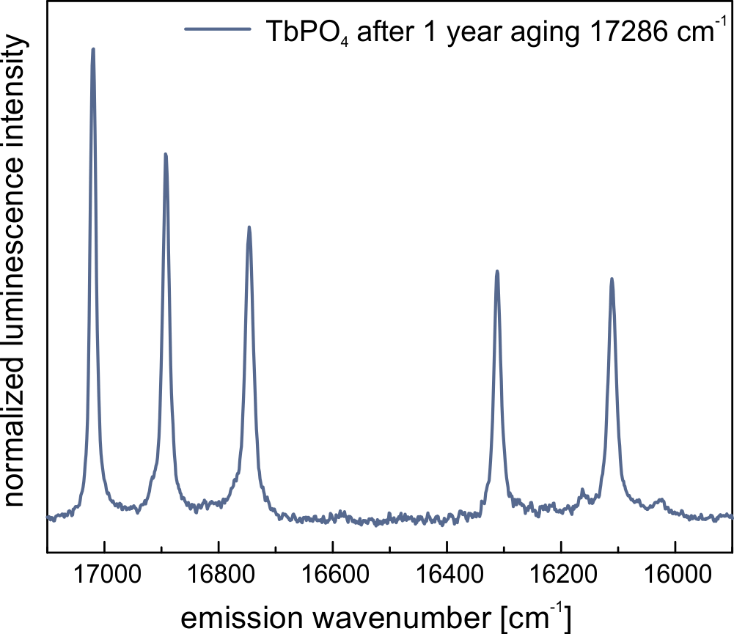


SI 3: Emission spectra of Eu^3+^‒doped TbPO_4_ after one year of aging, excited at 17268 cm^–1^.


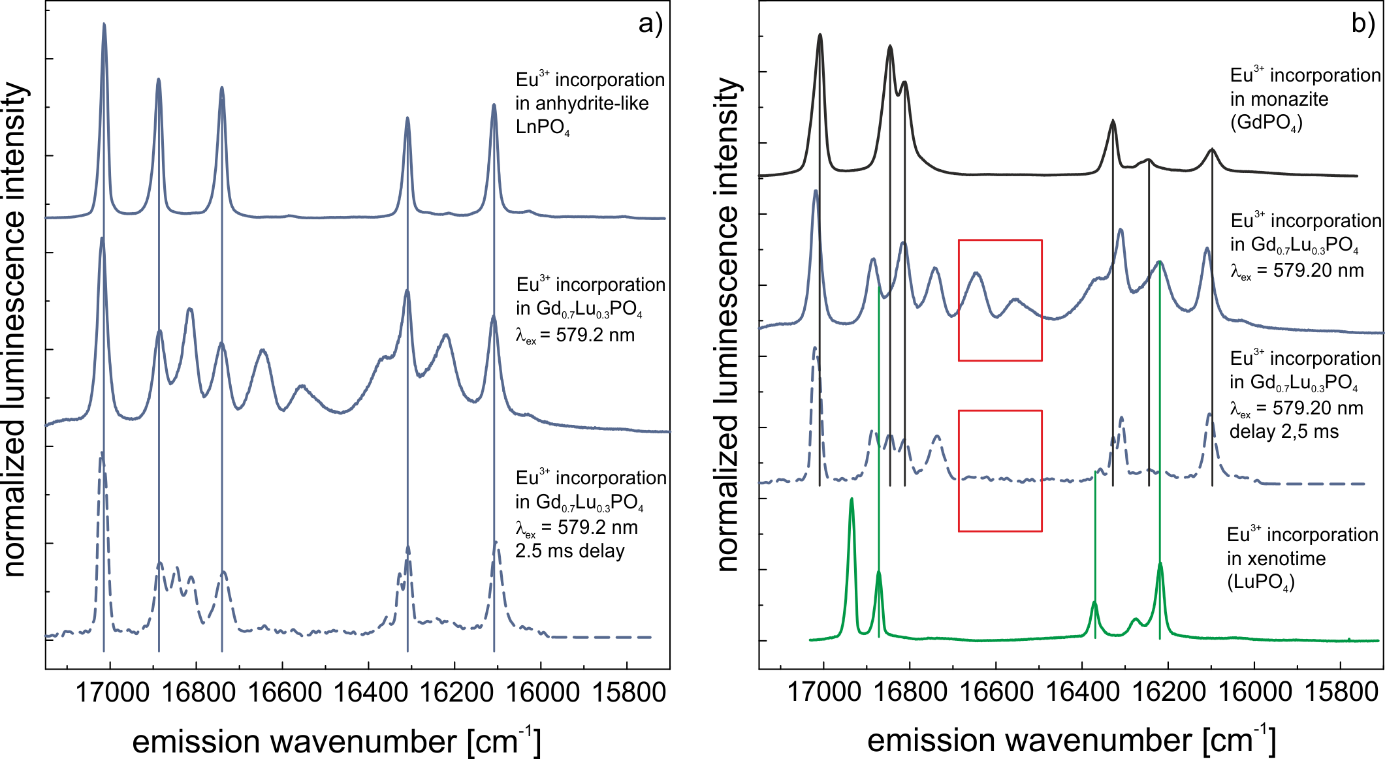


SI 4: Emission spectra of Eu^3+^ doped Gd_0.7_Lu_0.3_PO_4_ (λ_ex_ = 17265 cm^–1^, delay = 10 μs or 2.5 ms) compared with a) Eu^3+^ incorporation in an anhydrite–like phase and b) Eu^3+^ incorporation in monazite (black traces) and xenotime (green traces) phase. The red box indicates Eu^3+^‒emission signals that cannot be ascribed to any of the *Ln*PO_4_ phases.


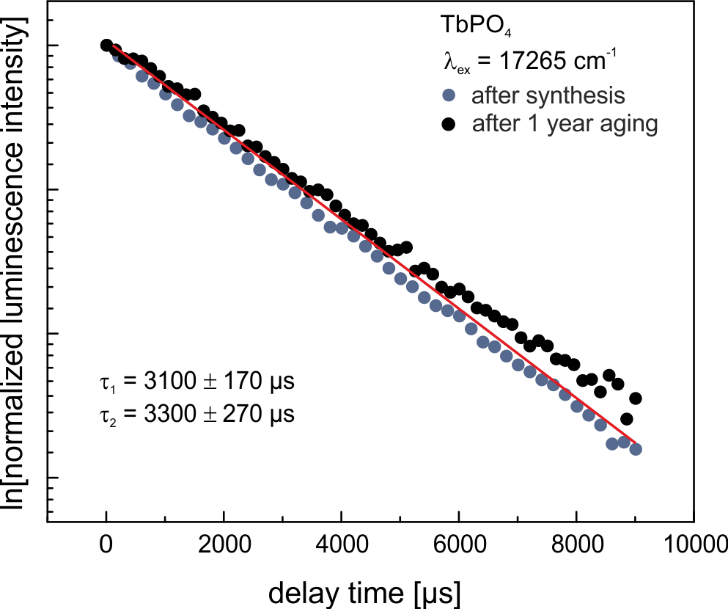


SI 5: Lifetimes of Eu^3+^–doped TbPO_4_ after synthesis (gray) and after one year of aging (black).


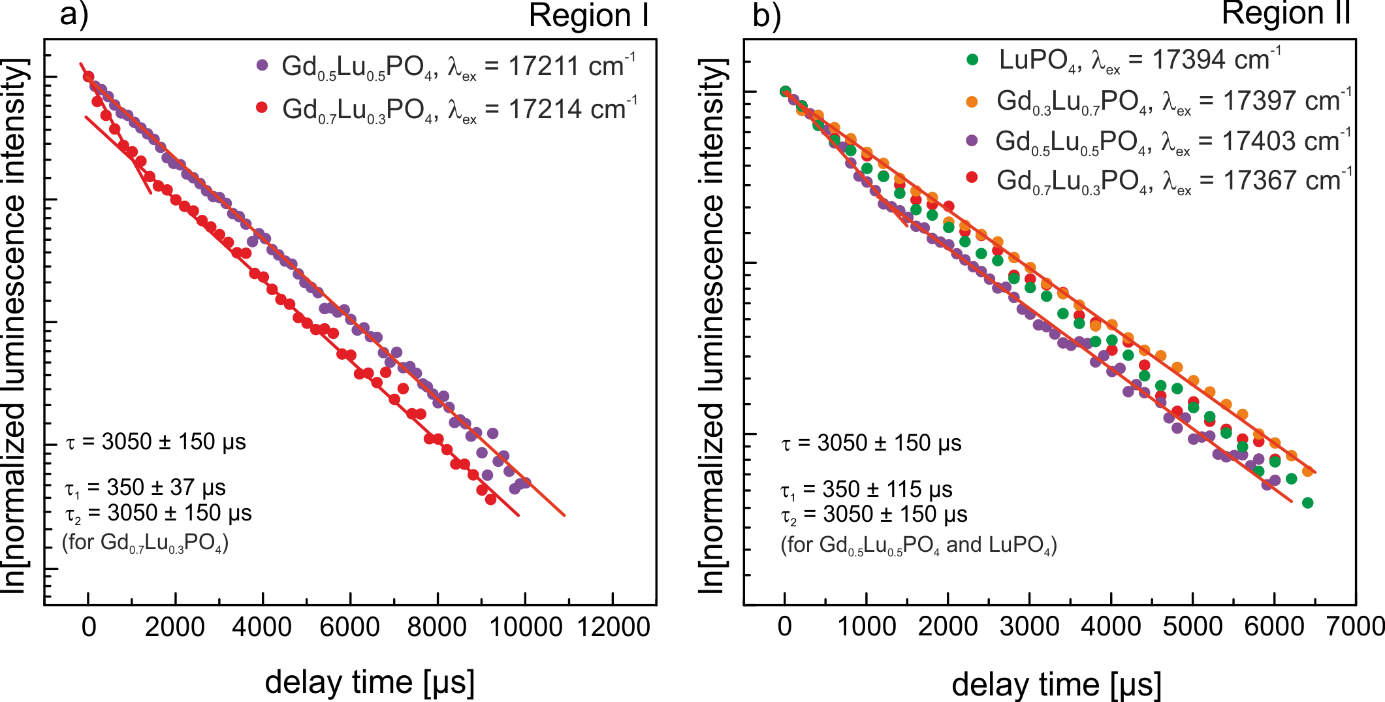


SI 6: Lifetimes of different Gd,Lu–solid solutions in a) Region I and b) Region II (right) after one year aging.


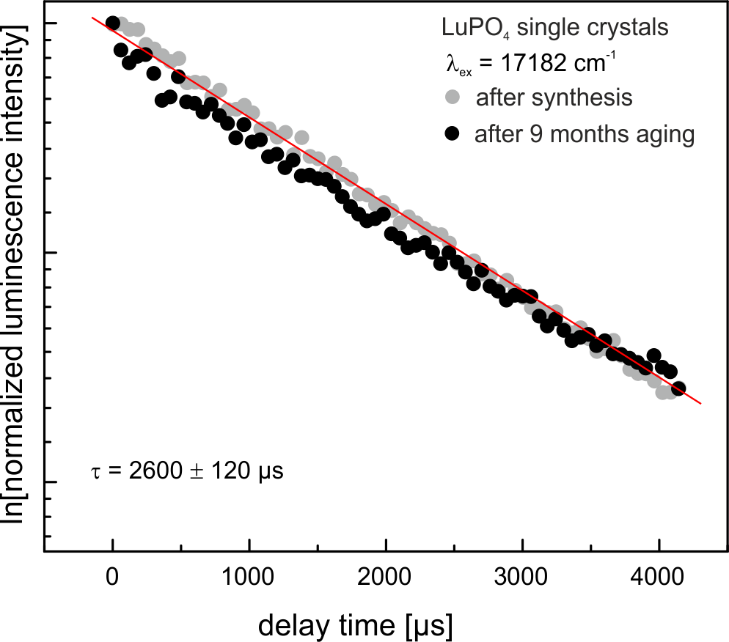


SI 7: Lifetimes of Eu^3+^‒doped LuPO_4_ single crystals direct after the synthesis (gray) and after 9 months of aging (black).


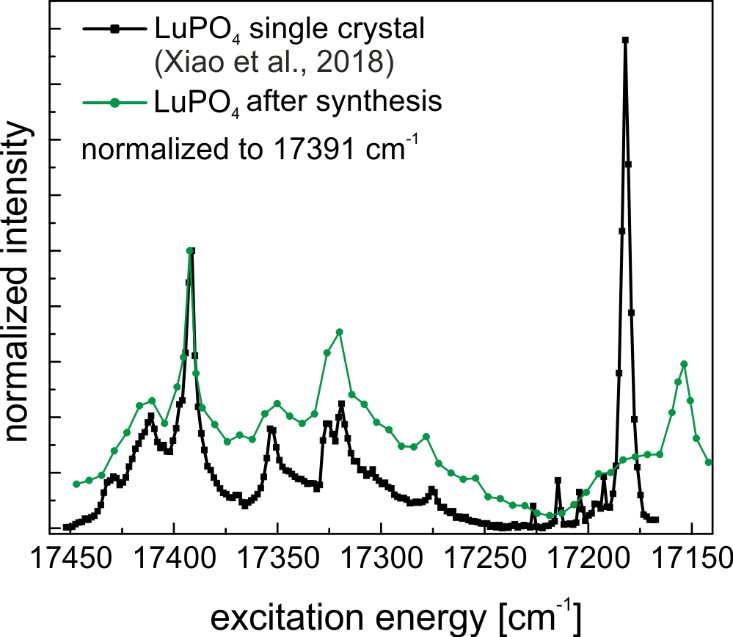


SI 8: Comparison of excitation spectra of LuPO_4_ powder and single crystal samples measured directly after synthesis. The single crystal data are taken from (Xiao *et al.*, 2018).

**References**

Junot, D.O., dos Santos, M.A.C., Antonio, P.L., Caldas, L.V., and Souza, D.N. (2014), Feasibility study of CaSO_4_: Eu, CaSO_4_:Eu, Ag and CaSO_4_:Eu, Ag (NP) as thermoluminescent dosimeters, Radiation Measurements, 71, 99-103, doi: 10.1016/j.jcis.2016.08.027.

Xiao, B., Lösch, H., Huittinen, N., and Schmidt, M. (2018), Local structural effects of Eu^3+^ incorporation into xenotime-type solid solutions with different host cations, Chemistry – A European Journal, 24(50), 13368-13377, doi: 10.1002/chem.201802841.
